# UBQLN2 Mediates Autophagy-Independent Protein Aggregate Clearance by the Proteasome

**DOI:** 10.1016/j.cell.2016.07.001

**Published:** 2016-08-11

**Authors:** Roland Hjerpe, John S. Bett, Matthew J. Keuss, Alexandra Solovyova, Thomas G. McWilliams, Clare Johnson, Indrajit Sahu, Joby Varghese, Nicola Wood, Melanie Wightman, Georgina Osborne, Gillian P. Bates, Michael H. Glickman, Matthias Trost, Axel Knebel, Francesco Marchesi, Thimo Kurz

**Affiliations:** 1 Institute of Molecular, Cell and Systems Biology, College of Medical, Veterinary and Life Sciences, Davidson Building, Henry Wellcome Lab of Cell Biology, University of Glasgow, G12 8QQ Glasgow, UK; 2 The MRC Protein Phosphorylation and Ubiquitylation Unit, The Sir James Black Centre, College of Life Sciences, University of Dundee, Dow Street, Dundee DD1 5EH, Scotland; 3 Newcastle University Protein and Proteome Analysis, Devonshire Building, Devonshire Terrace, Newcastle upon Tyne NE1 7RU, UK; 4 Department of Biology, Technion-Israel Institute of Technology, 32000 Haifa, Israel; 5 Department of Medical and Molecular Genetics, King’s College London, 8th Floor Tower Wing, Guy’s Hospital, Great Maze Pond, London SE1 9RT, UK; 6 School of Veterinary Medicine, College of Medical, Veterinary and Life Sciences, University of Glasgow, 464 Bearsden Road, Glasgow G61 1QH, UK

## Abstract

Clearance of misfolded and aggregated proteins is central to cell survival. Here, we describe a new pathway for maintaining protein homeostasis mediated by the proteasome shuttle factor UBQLN2. The 26S proteasome degrades polyubiquitylated substrates by recognizing them through stoichiometrically bound ubiquitin receptors, but substrates are also delivered by reversibly bound shuttles. We aimed to determine why these parallel delivery mechanisms exist and found that UBQLN2 acts with the HSP70-HSP110 disaggregase machinery to clear protein aggregates via the 26S proteasome. UBQLN2 recognizes client-bound HSP70 and links it to the proteasome to allow for the degradation of aggregated and misfolded proteins. We further show that this process is active in the cell nucleus, where another system for aggregate clearance, autophagy, does not act. Finally, we found that mutations in UBQLN2, which lead to neurodegeneration in humans, are defective in chaperone binding, impair aggregate clearance, and cause cognitive deficits in mice.

## Highlights

• UBQLN2 clears aggregates independent of autophagy via HSP70 and the proteasome
• A disease mutation in UBQLN2 prevents its binding to HSP70
• Mutant UBQLN2 is defective in clearance of aggregates in vivo
• UBQLN2 knockin mice develop cognitive impairment and brain pathology

## Graphical Abstract

**Figure:**
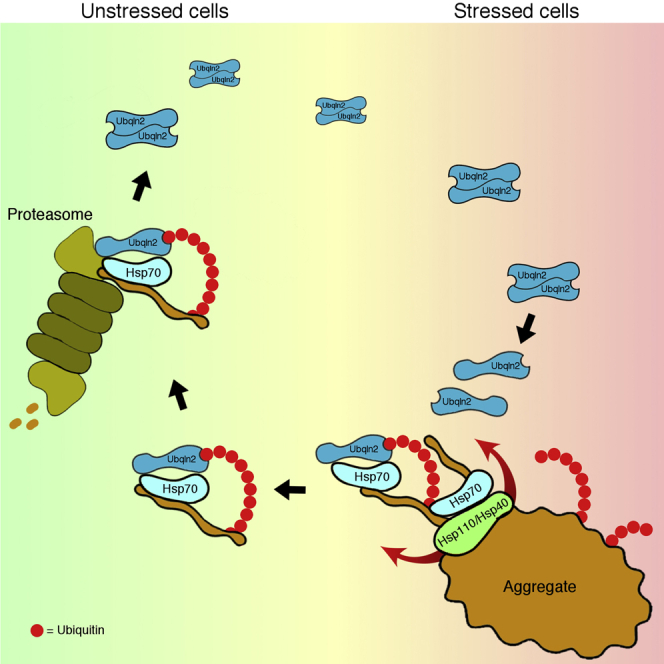

## Teaser

The proteasome teams up with partner proteins to clear tightly aggregated, potentially toxic species from the nucleus.

## Introduction

The modification of proteins with ubiquitin regulates most cellular pathways. A major role for ubiquitylation is to target proteins for degradation via the 26S proteasome, forming the so-called ubiquitin-proteasome system (UPS) (Glickman and Ciechanover, 2002). Ubiquitin chains are built on substrates by E3 ubiquitin ligases, which link the first ubiquitin via its C terminus to the ε-amino group of an internal lysine residue of the substrate, followed by the conjugation of subsequent ubiquitin moieties to a lysine of the preceding ubiquitin (Thrower et al., 2000, Shabek et al., 2012, Lu et al., 2015). Specificity in the UPS is largely mediated by the ∼600 E3 ubiquitin ligases that recognize their cognate substrates, but there is also selectivity on the level of delivery to the 26S proteasome, as ubiquitylated proteins are either directly recognized by the proteasome through stoichiometric subunits (RPN10 and RPN13) or through loosely associated shuttle factors, which link polyubiquitylated proteins and the proteasome to facilitate degradation. Budding yeast has three shuttles: Dsk2, Rad23, and Ddi1 (Verma et al., 2004, Elsasser et al., 2004). These have an N-terminal ubiquitin-like (UBL) domain, which interacts with the proteasome (Elsasser et al., 2002, Saeki et al., 2002), and a C-terminal ubiquitin-associated (UBA) domain, which binds polyubiquitylated proteins. They also all contain domains between the UBL and UBA domains, whose functions are largely unexplored. An important observation is that UBL-UBA domain proteins act as inhibitors of proteasomal degradation when overexpressed (Kleijnen et al., 2000, Chen and Madura, 2002, Funakoshi et al., 2002, Raasi and Pickart, 2003). It is thus vital to study these proteins at endogenous levels, as even small increases in their abundance inhibit proteasomal degradation (Verma et al., 2004). Similarly, overexpression of Dsk2 in yeast cells causes cell-cycle arrest and cell death (Matiuhin et al., 2008), and overexpressing UBQLN in *Drosophila* leads to photoreceptor neurodegeneration (Ganguly et al., 2008).

**Figure 1:**
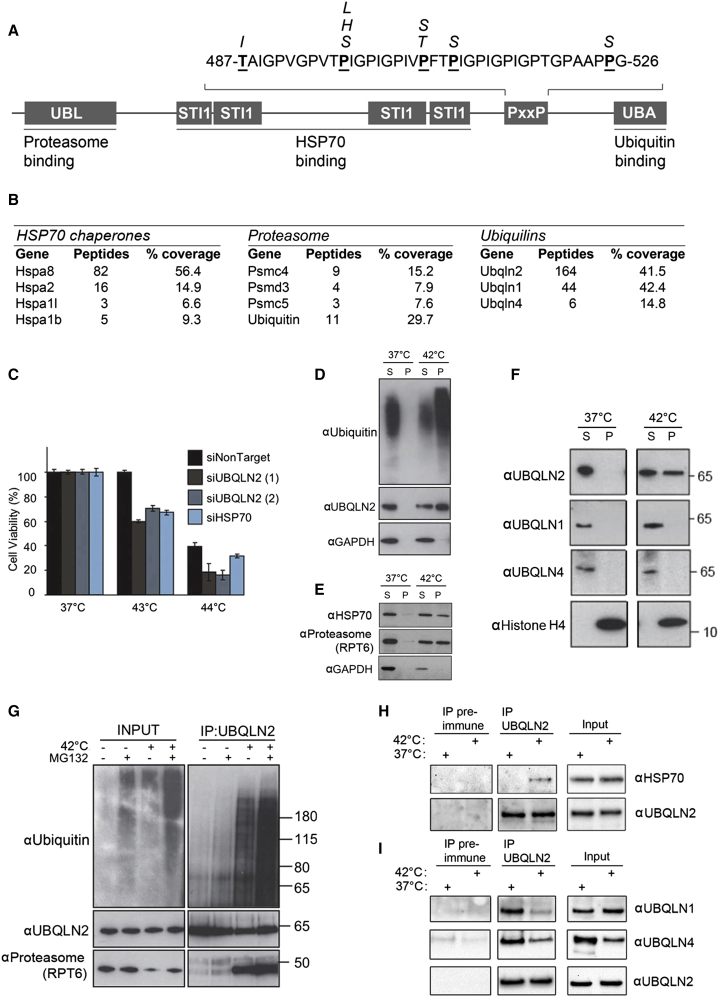
UBQLN2 Is Required for Cell Survival after Heat Shock (A) Schematic of the known domains of UBQLN2, their binding partners, and reported familial disease mutations shown in italics. (B) Binding partners of UBQLN2 that were identified by immunoprecipitation (IP) of UBQLN2 from mouse brain lysate followed by mass spectrometry. (C) Depletion of UBQLN2 by two independent siRNAs (72 hr) leads to cell death on heat stress. (D–F) UBQLN2, HSP70, and proteasome, but not UBQLN1 or UBQLN4, co-purify with insoluble ubiquitin-rich aggregates upon heat stress. (G–I) UBQLN2 inducibly interacts with proteasomes, ubiquitylated proteins, and HSP70 after heat shock and loses binding to UBQLN1 and UBQLN4. See also Figures S1 and S7.

Most vertebrates contain four homologs of the yeast protein Dsk2, which are named ubiquilin-1–4 (UBQLN1–4). While UBQLN1, 2, and 4 are expressed widely, UBQLN3 is restricted to testis (Marín, 2014). Part of the central region of UBQLN2 contains domains with homology to a heat shock binding protein called STI1, which binds Stch (HSP13), a protein similar to HSP70 (Kaye et al., 2000). UBQLN1, 2, and 4 each contain four such STI1 domains and can all interact with Stch (Lim et al., 2006, Wang et al., 2011, Rual et al., 2005), although the physiological role for this is currently unclear. UBQLN2 is mutated in familial cases of the protein folding disorder amyotrophic lateral sclerosis (ALS) (Deng et al., 2011), and intriguingly, all familial mutations cluster to the PXXP motif, which is unique to UBQLN2 and of unknown function (Deng et al., 2011, Fahed et al., 2014, Williams et al., 2012, Vengoechea et al., 2013) (Figure 1A).

The existence of shuttle factors is puzzling, and it is unclear why not all polyubiquitylated proteins are recognized by the intrinsic ubiquitin receptors of the proteasome. An attractive possibility is that shuttle factors add functionality to the proteasomal machinery to enable degradation of specialized substrates. We have explored this by studying the mammalian proteasome shuttle factor UBQLN2.

## Results

### UBQLN2 Is Required for Survival after Proteotoxic Stress

To better understand the role of UBQLN2 and its relevance to neurodegenerative disease, we isolated its binding partners from mouse brain using immunoprecipitation and mass spectrometry. UBQLN2 most evidently bound to HSP70-type chaperones, UBQLN1 and UBQLN4 (Figure 1B), and to a lesser extent to proteasomal subunits (Figure 1B).

Thus, UBQLN2 may be involved in the regulation of misfolded proteins. Indeed, UBQLN2 depletion by small interfering RNA (siRNA) caused hyper-sensitivity to heat shock, with a drop in cell viability comparable to the level observed after depletion of HSP70 (HSPA1A; Figure 1C).

**Figure S1:**
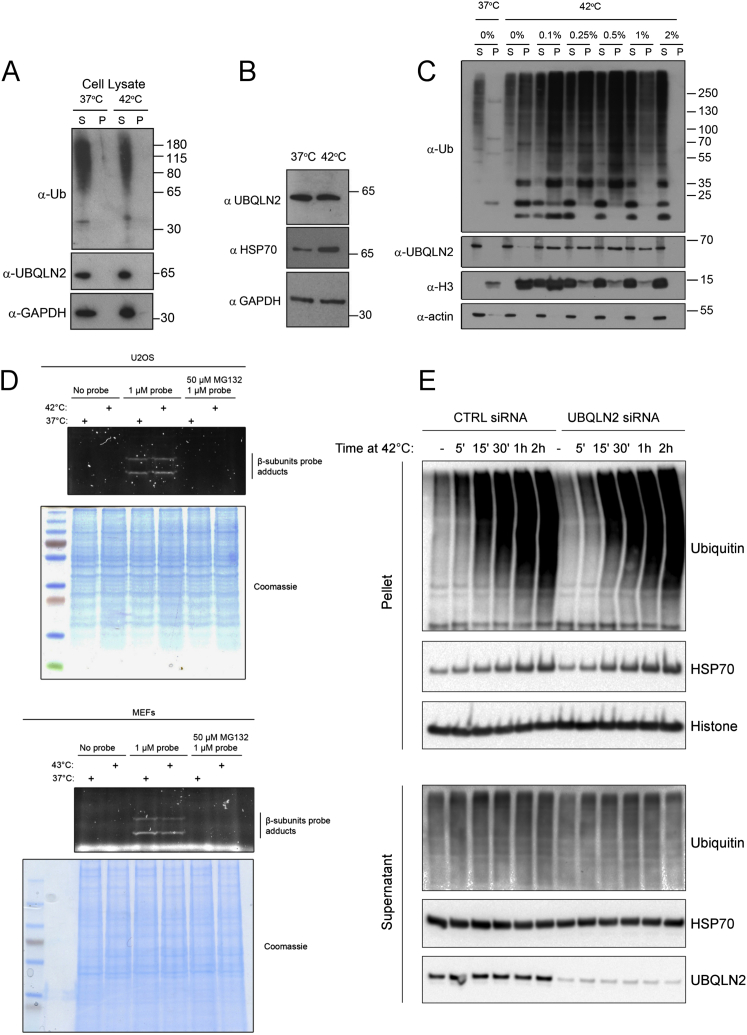
Heat Shock Generates Insoluble Ubiquitin-Positive Aggregates and Does Not Inactive Proteasomes, Related to Figure 1 (A) UBQLN2 is not pelleted when cells are heat shocked post lysis. Cell lysates were incubated at 37 or 42°C and then fractionated into soluble (S) and pellet (P) fraction. This indicates that UBQLN2 itself does not aggregate as a result of high temperature. (B) UBQLN2 levels are not upregulated in response to heat shock. HSP70 and GAPDH were used as a positive and negative controls, respectively. (C) Heat shock aggregates are insoluble in up to and including 1% SDS but are solubilized in 2% SDS. Blotting of soluble and pellet fractions with anti-ubiquitin and UQBLN2 antibodies confirmed dissolution of the aggregates in 2% SDS. (D) Proteasomes are active after heat shock. To confirm that proteasome activity was not affected by heat shock, we incubated U2OS and MEFs at the indicted temperatures for 2h. Cells were then harvested and cell lysates were incubated with the proteasome inhibitor MG132 or DMSO, followed by incubation with a fluorescent proteasome-activity probe, as indicated. The presence of fluorescently labeled beta-subunits at the same intensity under both heat stress and normal temperature, indicate that proteasome activity is not significantly affected by heat shock. (E) U2OS cells were treated with control or UBQLN2 siRNA and subjected to heat shock for the indicated times. Analysis of the pellet fraction revealed that insoluble ubiquitylated aggregates are generated within 5 min of heat shock, but that depletion of UBQLN2 does not noticeably alter the accumulation of these aggregates at any of the indicated time points.

Previous work showed that UBQLN2 binds to a range of protein aggregates in patient brains (Mori et al., 2012). We established that endogenous UBQLN2 similarly co-purifies with ubiquitylated insoluble protein aggregates generated by heat shock (Figure 1D), along with HSP70 and the proteasome (Figure 1E). Under non-stressed conditions (Figure 1D) or after heat shock of pre-lysed cells (Figure S1A), endogenous UBQLN2 is soluble, suggesting that UBQLN2 is not itself heat-unstable but rather actively recruited to aggregates. Interestingly, UBQLN1 and UBQLN4 remained soluble after heat stress (Figure 1F), which was surprising given their homology to UBQLN2.

Strikingly, we detected strongly increased binding of UBQLN2 to the proteasome and polyubiquitylated proteins after heat shock (Figure 1G), as well as enhanced binding to HSP70 (Figure 1H), suggesting the protein becomes activated under stress. UBQLN2 is not upregulated after heat shock (Figure S1B), indicating that it may instead be held in a repressed state under non-stressed conditions. Indeed, heat shock resulted in a loss of binding to other UBQLNs, consistent with a model where heterologous UBQLN complexes represent dormant reservoirs (Figure 1I).

### UBQLN2 Is a Proteasome Shuttle that Acts with the HSP70 System to Clear Aggregated Proteins

**Figure 2:**
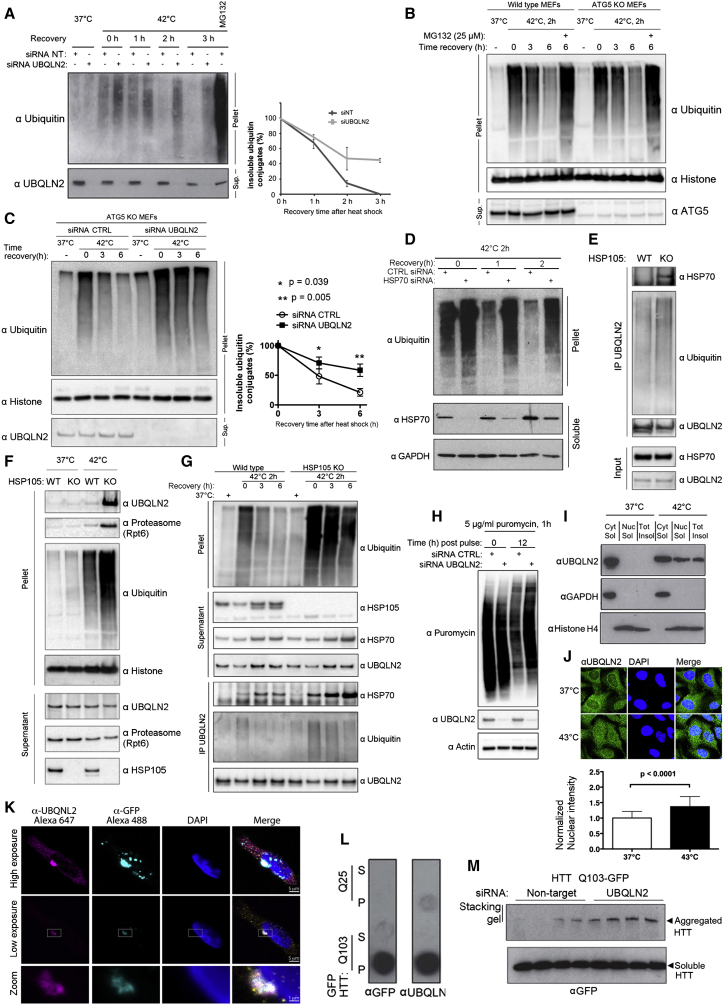
Heat Stress Activates UBQLN2 to Clear Aggregated Proteins (A) UBQLN2 depletion by siRNA leads to defective clearance of heat-shock-induced insoluble ubiquitin conjugates (left), and quantification of insoluble ubiquitin in the pellet (right) (n = 2). Error bars represent SEM. (B) Insoluble heat-shock-generated ubiquitin conjugates are cleared efficiently in ATG5 knockout (autophagy-deficient) MEFs in a proteasome-dependent manner. (C) UBQLN2 depletion in autophagy-deficient cells leads to attenuated clearance of heat-shock-induced insoluble ubiquitin conjugates. Quantification (n = 3) is shown (right). Error bars represent SD; statistical tests were two-tailed t tests. (D) HSP70 siRNA leads to a defective clearance of ubiquitylated aggregated proteins. Over time, the transcriptional heat shock response leads to increased levels of HSP70. (E) Increased interaction of UBQLN2 with HSP70 and ubiquitin was observed in HSP105 knockout (KO) MEF cells. (F) UBQLN2 and ubiquitin are more abundant in the pellet fraction after heat shock in HSP105 KO MEF cells. (G) HSP105 KO MEFs are deficient in clearing heat-shock-induced aggregates. In addition, increased binding of HSP70 and ubiquitin to UBQLN2 was detected. (H) Depletion of UBQLN2 by siRNA leads to defective clearance of puromycin-labeled truncated proteins. (I and J) UBQLN2 translocates to the nucleus after heat stress (see Figure S2A for fractionation protocol). Quantification of the normalized nuclear fluorescence intensity is shown (J, bottom) (n = 99 and 122 for 37°C and 43°C, respectively). Error bars represent SD. (K) UBQLN2 co-localizes with cellular HTT aggregates in HEK293 cells inducibly expressing pathological GFP-Huntingtin (HTTQ103). (L) UBQLN2 co-aggregates with pathological, but not non-pathological, GFP-Huntingtin, as shown by filter trap assay. (M) UBQLN2 depletion leads to increased HTT-Q103 aggregates, running in the stacking gel. Quadruplicate transfections are shown. See also Figures S2, S3, S4, and S7.

Heat shock generates aggregates of polyubiquitylated proteins insoluble in up to 1% SDS (Figure S1C), which are cleared by the proteasome (Figure 2A; Figure S1D). We found that siRNA depletion of UBQLN2 resulted in a pronounced defect in the clearance of heat-induced insoluble ubiquitin conjugates (Figure 2A) but did not affect their accumulation (Figure S1E), supporting a role of UBQLN2 in protein aggregate clearance. Large aggregates are thought to be degraded by a proteolytic mechanism called autophagy. Thus, we examined autophagy-defective atg5 knockout cells and found that these were just as capable as wild-type cells in clearing heat-induced aggregates (Figure 2B). In contrast, proteasomal inhibition led to a complete abrogation of clearance for both wild-type and atg5 knockout cells (Figure 2B). Clearance also required UBQLN2, as atg5 knockout cells where UBQLN2 was downregulated also no longer efficiently cleared the aggregates (Figure 2C). These results demonstrate that UBQLN2 mediates degradation of insoluble heat-shock-induced aggregates through the proteasomal pathway, independently of autophagy.

We next depleted HSP70 by siRNA and observed that HSP70 was also required to clear heat shock aggregates (Figure 2D). HSP70-mediated disaggregase activity requires the co-chaperone HSP110 (HSP105 in mice) (Nillegoda et al., 2015). To investigate whether UBQLN2 acts with the HSP70/HSP110 disaggregase pathway, we examined HSP110 (mHSP105) knockout mouse embryonic fibroblasts (MEFs) (Nakamura et al., 2008) and found that in these cells, interaction of both HSP70 and ubiquitin conjugates with UBQLN2 was increased even in the absence of heat stress (Figure 2E). This result suggested that in cells lacking HSP110, UBQLN2 becomes activated due to a higher aggregate load. In addition, heat shock induced a dramatic increase in the amount of UBQLN2, proteasome, and ubiquitin conjugates in the insoluble fraction of HSP110 knockout MEFs (Figure 2F), which also were impaired in their ability to clear heat shock aggregates (Figure 2G). These results demonstrate that UBQLN2 and the HSP70-HSP110 disaggregase act in the same pathway, and they explain how aggregates are processed by the chaperones prior to UBQLN2-mediated proteasomal degradation.

**Figure S2:**
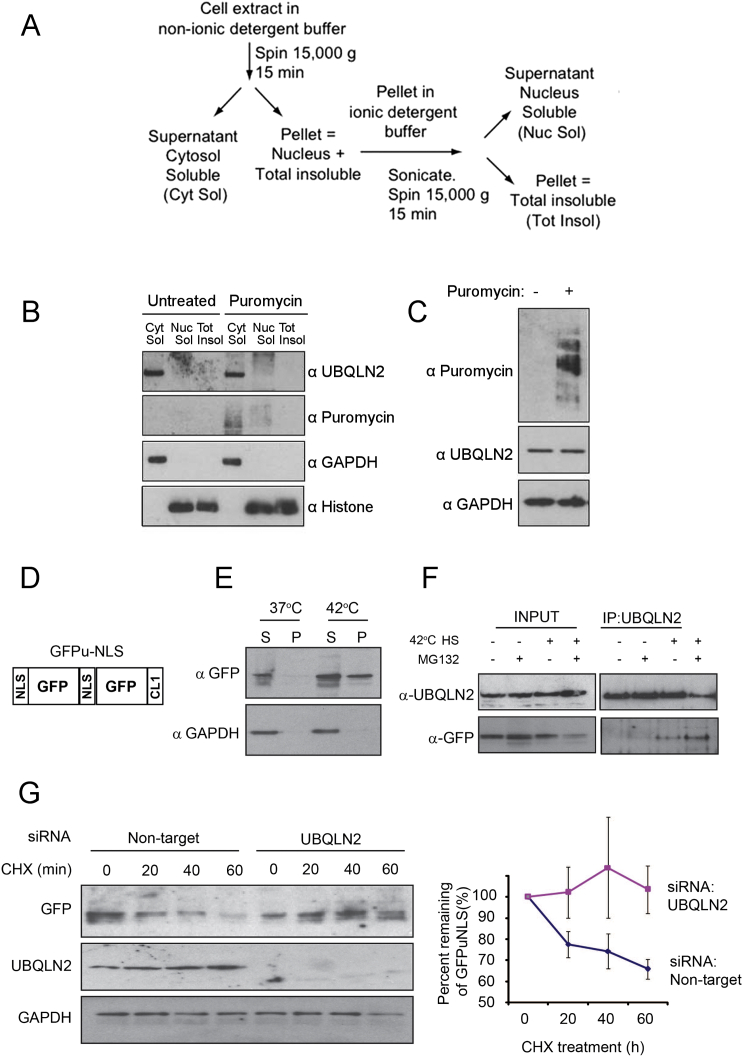
Puromycin Does Not Upregulate UBQLN2 Levels and UBQLN2 Clears Nuclear Aggregated GFP-u, Related to Figure 2 (A) Schematic representation on how the three fractions, total insoluble, nuclear soluble and total insoluble were generated. (B) Cells treated with puromycin were fractionated as indicated and treatment did not induce the nuclear localization of UBQLN2. (C) Puromycin treatment did not induce the upregulation of UBQLN2 protein. (D) Schematic showing the GFPu-NLS construct (Bennett et al., 2005). (E) HEK293 cells stably expressing GFPu-NLS were subject to heat shock for 2h at 42°C and fractionated into soluble and insoluble fractions. GFPu-NLS recruited to the insoluble fraction after heat shock indicating its heat-induced aggregation. (F) GFPu-NLS cells were subject to heat shock for 2h at 42°C and proteasome inhibition with 25 μM MG132 as indicated. UBQLN2 was immunoprecipitated and GFPu-NLS was found to co-immunoprecipitate only upon heat shock, consistent with UBQLN2 nuclear localization. Combined heat shock and proteasome inhibition increased the binding further. (G) GFPu-NLS cells were depleted of UBQLN2 or treated with a control non-targeting siRNA then treated with 50 μg/ml cycloheximide (CHX) for the indicated time to measure turnover. Turnover was quantified using data from three independent experiments. Error bars represent SE.

We next tested if UBQLN2 also mediates the degradation of unfolded proteins independent of heat stress. The antibiotic puromycin leads to the accumulation of unfolded nascent polypeptide chains (Eggers et al., 1997), and we found that UBQLN2 depletion impaired the clearance of these faulty translation products (Figure 2H), while UBQLN2 levels remained unchanged (Figure S2C).

Since many protein aggregates are found in the nucleus, where autophagy does not act, we next tested if UBQLN2 can enter the nucleus to clear protein aggregates. Using both biochemical fractionation (Figure 2I; Figure S2A) and immunofluorescence (Figure 2J), we found that UBQLN2 translocates into the nucleus upon heat stress, similar to HSP70 and other quality control components (Velazquez and Lindquist, 1984, Park et al., 2013). This did not happen using puromycin (Figure S2B), which generates unfolded proteins in the cytoplasm. To test if UBQLN2 clears nuclear substrates, we used cells stably expressing GFPu-NLS (Bennett et al., 2005), a model unfolded nuclear protein. Heat shock causes aggregation of GFPu-NLS (Figures S2D and S2E) and results in interaction of UBQLN2 with GFPu-NLS (Figure S2F), coinciding with nuclear translocation of UBQLN2. Moreover, the proteasomal degradation of GFPu-NLS after heat shock was dependent on UBQLN2 (Figure S2G), demonstrating that UBQLN2 can clear nuclear aggregates.

**Figure S3:**
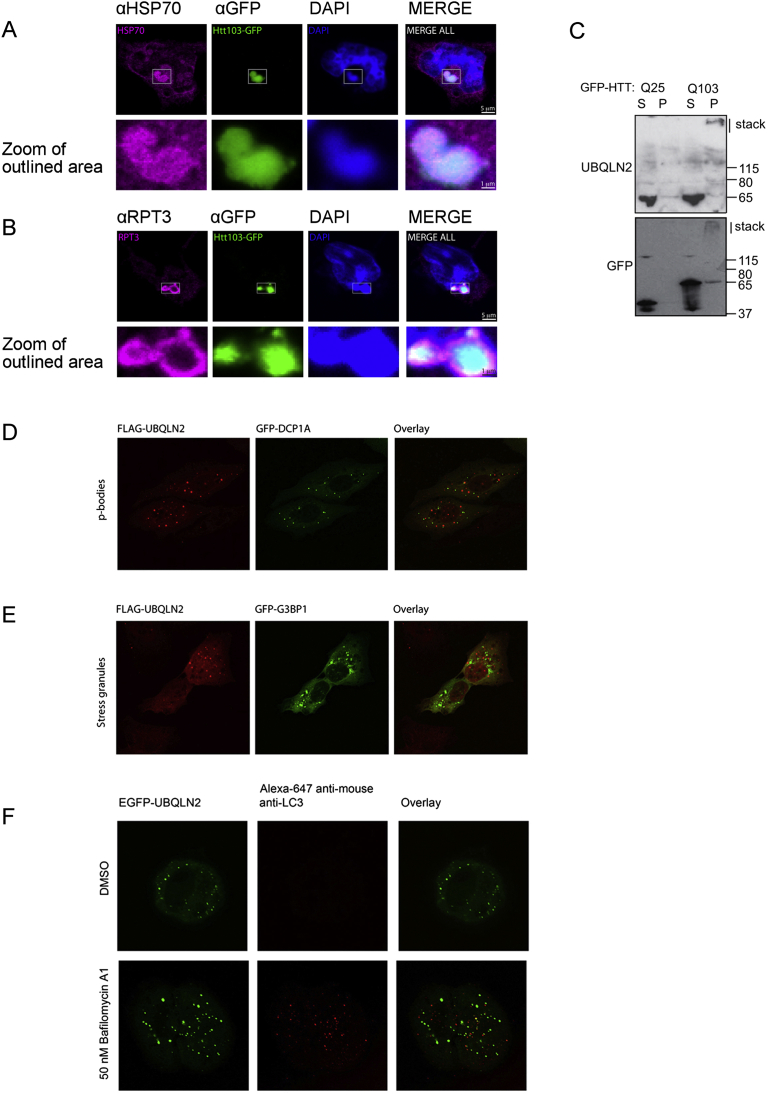
Proteasomes and HSP70 Co-localize with HTTQ103 Aggregates and Overexpressed UBQLN2 Form Cytosolic Foci, Related to Figures 2 and 3 (A and B) HEK293 cells expressing inducible HttQ103-GFP were stained with antibodies to (A) HSP70 and (B) proteasome subunit RPT3. Inclusion bodies were positive for booth HSP70 and the proteasome, the latter forming a ring around the perimeter of the inclusion. (C) Endogenous UBQLN2 co-aggregates with pathological Huntingtin (HTT-Q103) but not with non-pathological HTT-Q25. GFP-HTT Q25 or Q103 expression was induced in HEK293 cells, followed by cell harvesting and fractionation into soluble (S) and pellet (P) fractions. HTT-Q103 runs as high molecular weight aggregates present in the stacking gels for the pellet fraction, and endogenous UBQLN2 is observed to also be upshifted to the stacking gel. (D) The cytosolic foci visible on UBQLN2 overexpression do not co-localize with markers for p-bodies or stress-granules. (E) FLAG-UBQLN2 was transiently transfected into U2OS cells, with either GFP-DCP1A (p-body marker) or GFP-G3BP1 (stress granule marker). UBQLN2 was detected by indirect immunofluorescence to the FLAG-tag, using mouse monoclonal anti-FLAG antibody (SIGMA ALDRICH F3165), and an anti-mouse secondary Alexa Fluor 647 (Jackons 715-605-151). (F) Cytosolic UBQLN2 foci do not co-localize with the autophagosome marker LC3. U2OS cells stably expressing inducible EGFP-UBQLN2 were induced with 2 ng/ml doxycycline for 24 hr, then treated with 50 nM Bafilomycin A1 for 1 hr prior to fixation and staining. LC3 staining was performed using a mouse monoclonal anti-LC3 (MBL M152-3). Secondary antibody was anti-mouse Alexa Fluor 647 (Jackson 715-605-151). Vehicle control was DMSO.

We next examined the requirement of UBQLN2 for the clearance of a pathological Huntingtin fragment (HTTQ103), as UBQLN2 has been described to bind to aggregates in mouse models and patients with Huntington’s disease (HD) (Doi et al., 2004, Rutherford et al., 2013). We detected recruitment of endogenous UBQLN2 to HTT aggregates (Figure 2K), alongside HSP70 and the 26S proteasome (Figures S3A and S3B). We next found that the insoluble fraction from cells expressing GFP-HTTQ103, but not non-pathological GFP-HTTQ25, is retained on a filter trap alongside endogenous UBQLN2 (Figure 2L). HTTQ103 aggregates are retained in the stacking gel in SDS-PAGE, where we found that they also trap endogenous UBQLN2 (Figure S3C), and downregulation of UBQLN2 led to increased HTTQ103 aggregation (Figure 2M). Thus, UBQLN2 regulates degradation of model and disease-linked aggregation-prone proteins. Importantly, we demonstrate that the UBQLN2/HSP70/26S-proteasome pathway can clear aggregates in the nucleus.

### UBQLN2 Mutations Do Not Lead to UBQLN2 Aggregation

**Figure 3:**
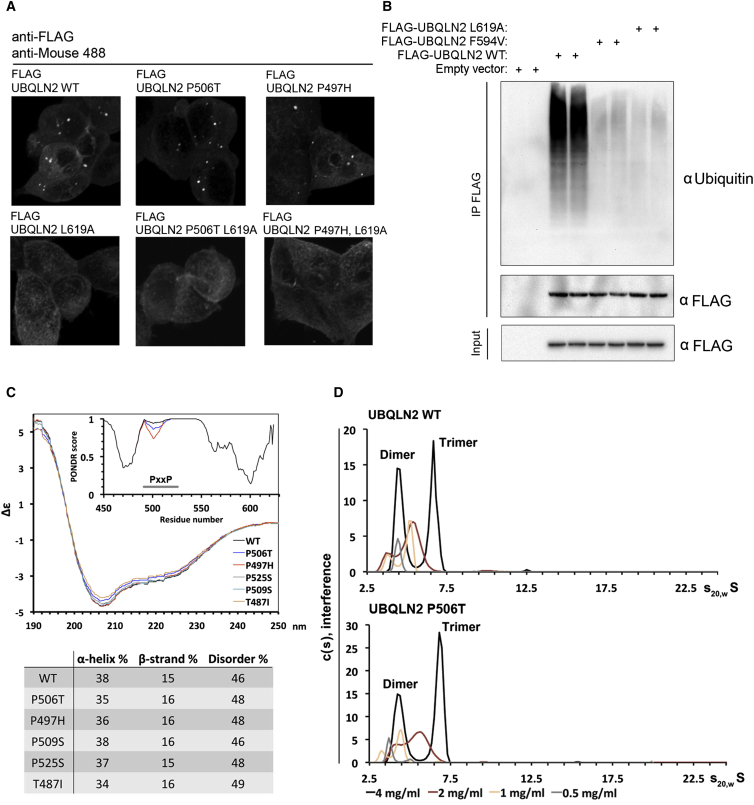
UBQLN2 Mutations Do Not Cause Protein Aggregation (A) Inducible HEK293 cells stably overexpressing the indicated FLAG-UBQLN2 exhibit cytosolic foci for both the wild-type (WT) and P506T mutant. The L619A ubiquitin non-binding point mutation abrogates foci formation for WT and P506T mutant. (B) UBQLN2 point mutants (F594V and L619A) are defective in polyubiquitin binding. (C) Circular dichroism performed on pure wild-type and mutant protein. PONDR prediction (inset) results in a small decrease of disorder for PXXP mutant proteins (WT, P506T, and P497H shown). Experimentally, no difference is seen in the amount of disorder and secondary structure for the mutants. (D) Purified UBQLN2 was analyzed by analytical ultracentrifugation at different concentrations, showing dimer and trimer peaks for both WT and mutant protein. See also Figures S3 and S4.

**Figure S4:**
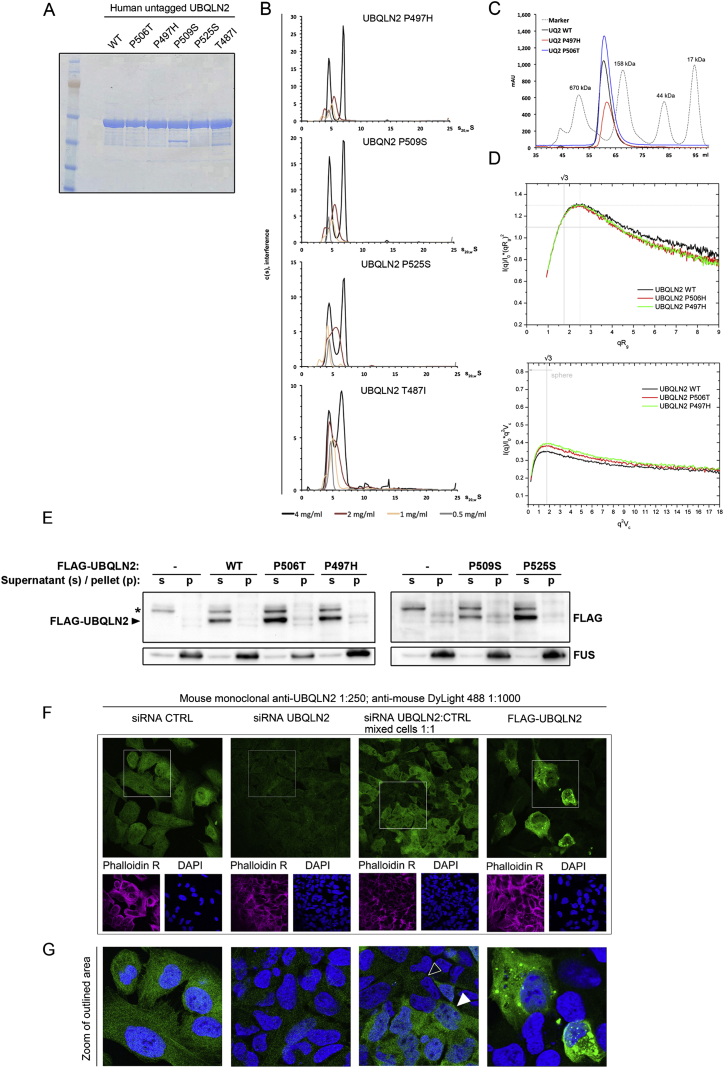
UBQLN2 Does Not Form Aggregates, and the Antibody Used for UBQLN2 Immunofluorescence Is Specific for UBQLN2, Related to Figures 2 and 3 (A) Coomassie stain of bacterially expressed and purified, untagged UBQLN2 wild-type and mutant proteins. (B) Analytical ultracentrifugation was performed to investigate differences in oligomerization or aggregation for purified UBQLN2. Additional mutants shown here to support results in main Figure 3. No significant amount of aggregated protein was detected for any mutant, and no differences in dimerization or trimerisation were observed. (C) Analytical gel filtration of UBQLN2 WT, P506T and P497H show a single sharp peak migrating at an apparent molecular weight above 158 kDa, without any indication of additional UBQLN2 species or aggregated material. (D) UBQLN2 P506T and P497H mutants are more compact particles than WT – flexibility analysis based on small-angle X-ray scattering experiments. The radius of gyration based dimensionless Kratky plot (top panel) has a characteristic shape for partially disordered protein containing both ordered and disordered fragment(s) – the peak of the curve (dotted gray line) is shifted from a position characteristic for globular folded protein (solid gray line). At the same time the plot demonstrates that WT contains more disorder (the right wing on the WT curve is slightly lifted comparing with P506T and 497H). The protein concentrations were 4.67, 4.03, 4.91 mg/ml for WT, P506T and 497H respectively. The volume-of-correlation based dimensionless Kratky plot (bottom panel) gives a more in-depth analysis and reveals the increase in volume-to-surface ratio for the P506T and 497H mutants, indicating more compact particles compared to WT UBQLN2 (the maximal possible volume-to-surface ratio of 0.82 is for a sphere; see arrow). (E) Mutations in UBQLN2 do not cause the protein to become insoluble in cells. FLAG-tagged wild-type and mutant UBQLN2 were overexpressed, and cells were fractionated into 1% NP-40 soluble (S) and insoluble pellet (P) fractions, and detected with FLAG-HRP conjugated antibody (SIGMA ALDRICH A8592). No difference in distribution as compared to the wild-type was seen for any of the mutants. FUS was used as a marker for the pellet. (F and G) Validation of UBQLN2 antibody for staining of endogenous UBQLN2 in U2OS cells. U2OS cells were transfected with control siRNA or siRNA targeting UBQLN2. 72h post-transfection, cells were trypsinised, and seeded on glass slides for microscopy. Cells were either seeded as separate groups (i.e., control and UBQLN2 siRNA) or mixed 1:1 and seeded together (third panel from the left). As a separate control, cells were transfected with plasmid encoding for FLAG-tagged UBQLN2 (right-most panel only). These cells show large UBQLN2 foci not present at endogenous levels. Cells were stained using the mouse monoclonal anti-UBQLN2 6H9 (Novus NBP2-25164), at 1:250 in 2% BSA PBS for 1h. Secondary antibody was goat Anti-Mouse DyLight 488 (Abcam ab96871). Knockdown of UBQLN2 can be clearly seen to decrease the signal, indicating that the antibody is specific to UBQLN2. (G) displays zoom of the indicated areas in. For the mixed cells (third panel from the left) a white arrowhead indicates a cell transfected with control siRNA and a black arrowhead a cell transfected with UBQLN2 siRNA.

We next examined the disease-linked mutations of UBQLN2 found in patients with familial ALS. Previous reports have suggested that both wild-type (WT) and mutant UBQLN2 aggregate, as exogenous expression leads to their localization to cytoplasmic foci similar in appearance to aggregates (Deng et al., 2011, Osaka et al., 2015). Indeed, exogenously expressing UBQLN2 in cells causes formation of cytosolic foci (Figure 3A), but no gross differences in size or number of foci were seen for mutant UBQLN2 (P506T, P497H) (Figure 3A). Importantly, mutating the UBA domain (L619A) to abolish ubiquitin binding (Figure 3B) leads to complete exclusion of both WT and mutant forms of UBQLN2 from the foci (Figure 3A), strongly suggesting the foci are not misfolded UBQLN2. The foci do not co-localize with as P bodies, stress granules (Figures S3D and S3E), or autophagosomes (Figure S3F). Furthermore, UBQLN2 foci formation does not render UBQLN2 insoluble, as UBQLN2 (WT) and five disease-linked mutants remained soluble when overexpressed in HEK293 cells (Figure S4E). Importantly, endogenous UBQLN2 is diffusely cytosolic (Figure 2J; Figure S4F).

Next, we used purified UBQLN2 to investigate the biophysical properties of the WT and mutant proteins (Figure S4A). Small angle X-ray scattering (SAXS) experiments using WT and two mutant forms of UBQLN2 (P506T and P497H; Figure S4A) indicated that the mutations reduce the flexibility of the protein (Figure S4D). Based on circular dichroism measurements, there are no gross differences in secondary structure for any tested mutant (Figure 3C). Using analytical ultracentrifugation, we detected that both WT and mutant UBQLN2 forms dimers and trimers in a concentration-dependent manner but no higher-number oligomers or aggregates, which we also confirmed by size exclusion chromatography (Figure 3D; Figures S4B and S4C).

### Disease-Linked UBQLN2 Mutation Impedes Binding to HSP70 Chaperones and Sensitizes Cells to Protein Folding Stress

**Figure 4:**
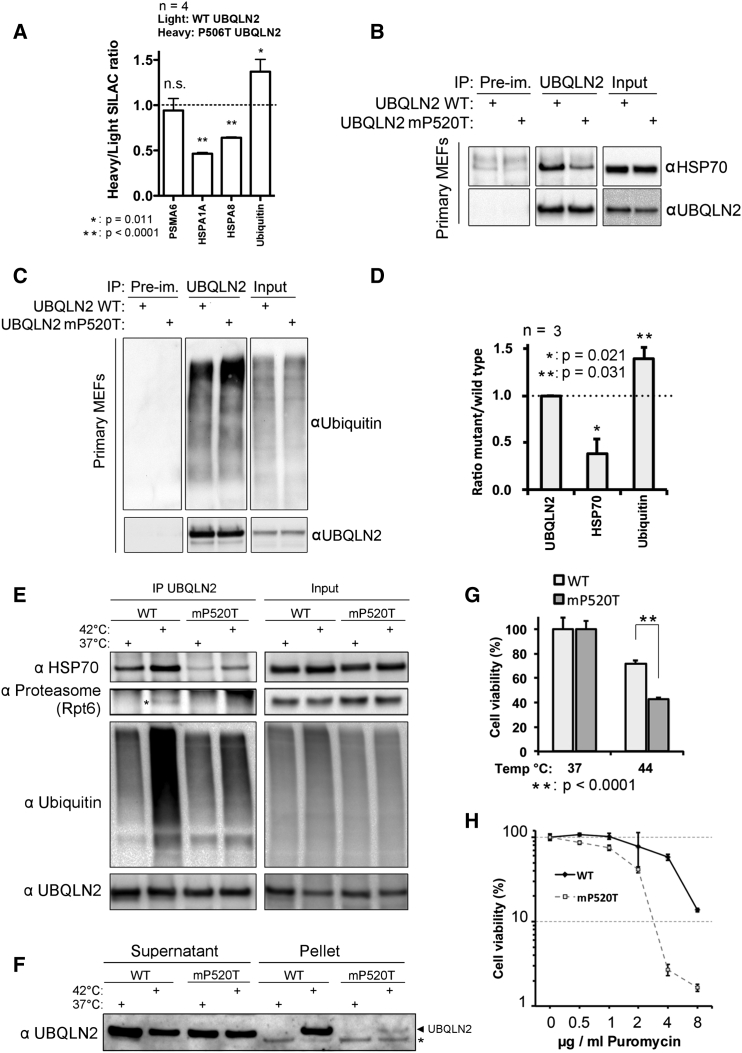
Disease Mutant UBQLN2 Loses Binding to HSP70 and Sensitizes to Protein Misfolding Stress (A) SILAC proteomics was performed on FLAG IP from cells stably expressing inducible FLAG-UBQLN2 WT or P506T. Interaction with proteasomal subunits (PSMA6 shown) is unaffected by the mutation, UBQLN2 P506T binding to HSP70 family members (HSPA1A, HSPA8) is significantly lower (p < 0.0001), and binding to ubiquitin is significantly higher (p = 0.011). Asterisks indicate a statistically significant difference from a SILAC ratio of 1 (two-tailed single-value t test). (B–D) Decreased binding to HSP70 and increased binding to ubiquitin was confirmed by UBQLN2 IP from wild-type and mP520T (equivalent to human P506T) primary male mouse embryonic fibroblasts (MEFs), derived from littermate embryos. HSP70 (B) and ubiquitin (C) were detected by western blot. (C) Quantification of mutant/wild-type signal ratio for co-immunoprecipitated HSP70 and ubiquitin. Asterisk indicates a statistically significant difference from a mean ratio of 1 (two-tailed single-value t test). (E) Stress-induced binding to HSP70, ubiquitin and proteasomes is defective for mutant UBQLN2. Asterisk indicates Rpt6 (proteasome). (F) Mutant UBQLN2 is defective in association to heat shock induced aggregates. Asterisk indicates a non-specific band. (G) mP520T MEFs are hypersensitive to heat shock as compared to WT counterparts. (H) mP520T MEFs are hypersensitive to 20-hr puromycin treatment at the indicated concentrations. Error bars represent SD. Statistical test was a two-tailed t test. See also Figures S5, S6, and S7.

**Figure S5:**
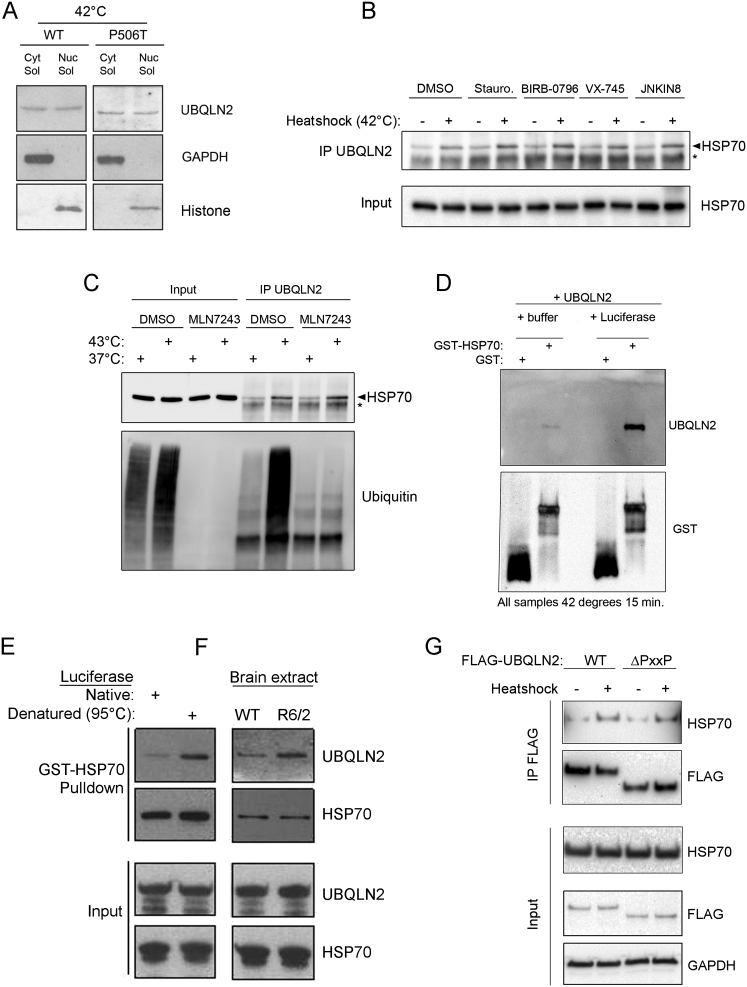
Nuclear Translocation of UBQLN2 Is Unaffected by Disease Mutation, and HSP70 Clients Induce HSP70-UBQLN2 Interaction, Related to Figures 4 and 5 (A) Wild-type of P520T knock-in MEFs were heat shocked and fractionated as indicated and no difference was observed in the nuclear localization as a result of the disease mutation. (B) HEK293 cells were treated with the broad spectrum kinase inhibitor Staurosporine (1 μM) or the p38 (BIRB-0796 and VX-745; 1 μM) and JNK (JNKIN8; 10 μM) kinase inhibitors for 1h prior to heat shock and showed that kinase signaling is not regulating the inducible interaction of HSP70 and UBQLN2. (C) HEK293 cells were treated with the ubiquitin E1 inhibitor MLN7243 (10 μM) for 1h prior to heat shock and demonstrated that ubiquitylation or ubiquitin signaling is not involved in regulating the inducible interaction between HSP70 and UBQLN2. (D) UBQLN2 does not bind non-specifically to GST in the presence or absence of denatured luciferase. GST or GST-HSP70 and purified UBQLN2 was incubated at 42°C in the presence or absence of Luciferase, as indicated. This was followed by GST pulldown, and Western blot for associated UBQLN2. (E) Luciferase was denatured at 95°C for 5 min and found to stimulate the binding of untagged recombinant UBQLN2 to GST-HSP70 upon GST-pulldown, unlike native luciferase. (F) R6/2 brain extracts but not WT brain extracts were found to be able to stimulate the interaction of recombinant untagged UBQLN2 with GST-HSP70 in GST pulldown experiments. (G) HEK293 cells stably expressing inducible UBQLN2 WT or PXXP deletion mutants were found to both equally interact with endogenous HSP70 after heat shock, indicating that the PXXP motif does not directly mediate the interaction.

**Figure S6:**
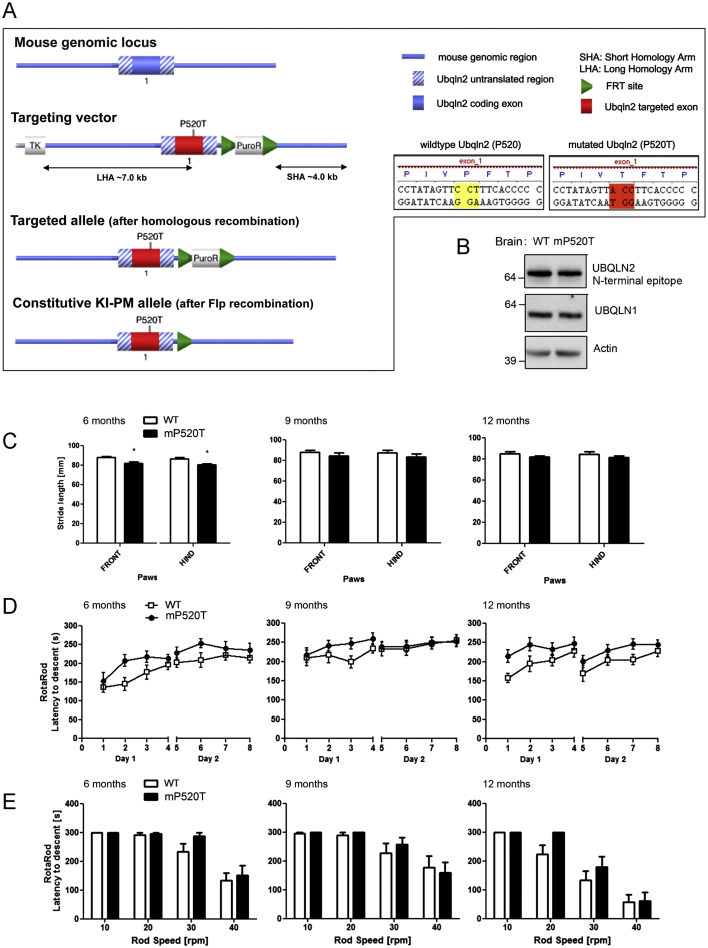
Generation of a Constitutive Knock-in Mouse Model and Locomotor Tests of a Male Cohort, Related to Figures 4, 5, and 6 (A) Targeting strategy used to generate the UBQLN2 P520T knock-in mice. (B) Western blot of brain extracts showing that UBQLN2 levels are expressed at the same level in WT and UBQLN2 knock-in male mice (expressing one copy each of UBQLN2 due to being X-linked). (C) Gait analysis in the mP520T mouse model. Gait analysis was performed at 6, 9 and 12 months of age, showing a marginal, but significant decrease in stride length at 6 months of age for the mutant animals. At 9 and 12 months the trend persists. Habituation to handling/runway corridor was followed by assessment of gait by painting of front and hind paws. Gait parameters including stride length and width between paws was analyzed manually from the paw print records. (D) Accelerating rotarod tests showed no impairment in motor function for UBQLN2 mP520T animals at any age. The animals performed 8 trials (4 trials on day 1 and a further 4 trials on the following day). On each trial the mouse was placed on the RotaRod and the rod accelerates from a speed of 5 rpm up to 45 rpm, with a maximum trial time of 5 min. (E) Fixed speed rotarod tests showed no impairment in motor function for UBQLN2 mP520T animals at any age.

As disease-linked mutant UBQLN2 did not aggregate, we next used stable isotope labeling with amino acids in cell culture (SILAC) proteomics to investigate changes in the interactome of cells stably expressing inducible WT or mutant UBQLN2. We found that disease-linked UBQLN2 (P506T) showed decreased binding to HSP70 chaperones and increased binding to ubiquitin (Figure 4A). We next generated a mouse knockin of the equivalent human P506T mutation (mP520T) and confirmed these changes at the endogenous level using primary MEFs from male mice (UBQLN2 is X linked) (Figures 4B–4D; Figures S6A and S6B). Strikingly, the binding of UBQLN2 to HSP70, ubiquitylated substrates and the proteasome after heat shock was strongly attenuated for mutant UBQLN2 (Figure 4E). Also, while the heat-shock-induced nuclear translocation of mutant UBQLN2 (mP520T) was unaffected (Figure S5A), it was strongly impaired in its recruitment to aggregates (Figure 4F), and cells expressing UBQLN2 (mP520T) were hypersensitive to both heat shock and puromycin stress compared to their wild-type littermate counterparts (Figures 4G and 4H). Together, these data suggest that the disease-linked forms of UBQLN2 are loss-of-function mutations.

**Figure 5:**
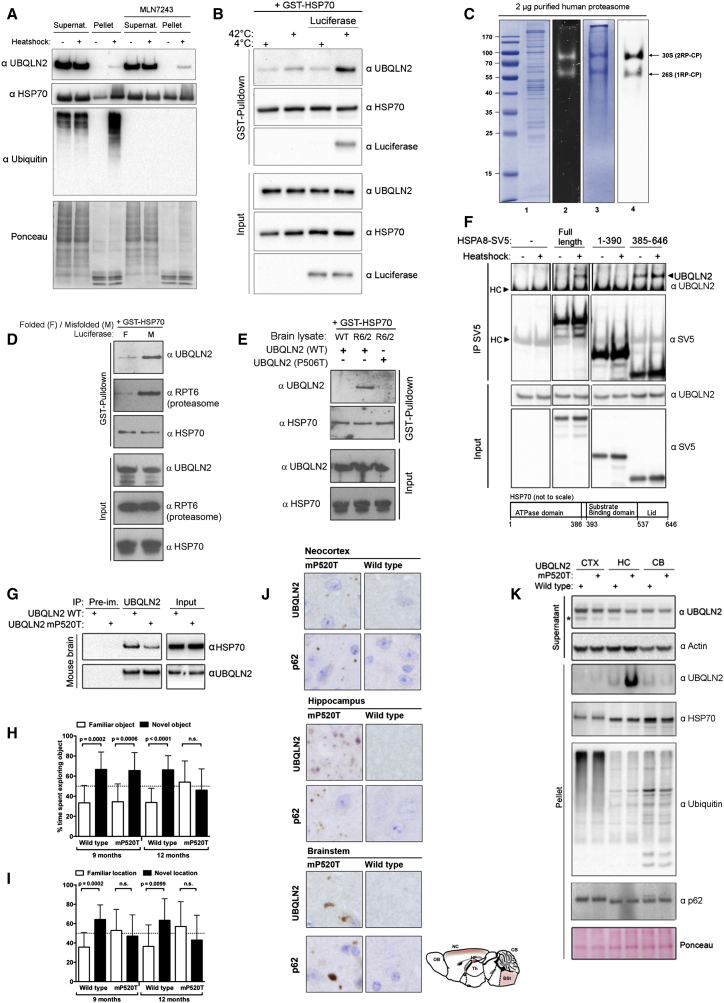
HSP70 Client Interaction Drives UBQLN2-HSP70 Binding (A) UBQLN2 association to heat-shock-induced pelleted proteins is independent of ubiquitin. Cells were treated with the ubiquitin E1 inhibitor MLN7243, heat shocked, and fractionated into supernatant and pellet. (B) Presence of HSP70-client induces UBQLN2-HSP70 interaction in vitro. Reaction components were mixed and incubated at the indicated temperature, followed by pull-down of GST-HSP70. (C) Purified human 26S proteasome. Lane 1, Coomassie staining of 2 μg purified human proteasome; lanes 2–4, in-gel LLVY-AMC (N-succinyl-leucine-leucine-valine-tyrosine-7-amino-4-methylcoumarin) chymotrypsin activity of 2 μg human proteasome, Coomassie staining, and immunoblot with anti-Rpt5 antibody in 4% native-PAGE, respectively. (D) Heat-denatured (95°C) or native recombinant luciferase was added to the other reaction components, followed by GST-HSP70 pull-down. (E) Pathological Huntingtin aggregates induce binding of GST-HSP70 to purified wild-type, but not mutant (P506T), UBQLN2 in vitro. Brain extract from wild-type or R6/2 mice was spiked into the reaction mix, followed by GST-HSP70 pull-down and analysis of UBQLN2 binding. (F) UBQLN2 binds to the C-terminal domain of HSP70. IP of HSPA8-SV5 mutants expressed in HEK293 cells and detection of endogenous UBQLN2. Cells were heat shocked as indicated. Schematic shows the HSP70 domains. (G) Mutant UBQLN2 shows reduced binding to HSP70 in knockin mouse brain. (H and I) The UBQLN2 mP520T knockin mutation leads to cognitive impairment in aged mice. Male mice (n = 11 of each genotype) were aged and tested in novel-object and novel-place recognition tests. Error bars represent SD. Statistical tests were two-tailed t tests. (J) Aged UBQLN2 mP520T knockin animals have UBQLN2- and p62-positive inclusion body pathology. Brains from aged (15- to 18-month-old) mice were subjected to immunohistochemistry (IHC) for UBQLN2 and p62 (n = 6 per genotype). Red shading in schematic shows areas of inclusion pathology. (K) Mutant UBQLN2 is specifically present in the pellet from hippocampal lysates in aged (15- to 18-month-old) knockin mice. Isolated neocortex (CTX), hippocampus (HC), and cerebellum (CB) were separated into NP40-soluble and insoluble fractions. Asterisk indicates an unspecific band. See also Figures S5, S6, and S7.

Since binding of UBQLN2 to HSP70 was unaffected by inhibiting stress inducible kinases or the ubiquitin E1 (Figures S5B and S5C) and recruitment of UBQLN2 to the insoluble fraction was also independent of ubiquitylation (Figure 5A), binding of UBQLN2 to HSP70 may in turn depend on client binding to HSP70.

To test this, we used an in vitro system to examine the effect of protein aggregates on the UBQLN2-HSP70 interaction. Strikingly, the interaction between HSP70 and UBQLN2 was only induced when reactions also contained HSP70 client in the form of either mildly denatured (42°C for 30 min; Figure 5B; Figure S5D) or strongly denatured (95°C for 5 min; Figure S5E) recombinant luciferase. We next tested if the presence of HSP70 client would also result in the recruitment of purified human proteasomes (Figure 5C). Indeed, the interactions among HSP70, UBQLN2, and proteasomes in vitro were strongly induced by the addition of denatured luciferase, demonstrating that the presence of substrate leads to the formation of degradation complexes (Figure 5D).

We next asked if a relevant pathological aggregate would have the same effect on HSP70/UBQLN2 complex formation. For this, we added small amounts of brain extracts from wild-type or R6/2 HD model mice (Mangiarini et al., 1996) to the in vitro interaction experiments and found that only the R6/2 extract triggered the interaction between HSP70 and UBQLN2 (Figure 5E; Figure S5F). This effect was seen with WT UBQLN2, but strikingly not with the disease-linked UBQLN2 (P506T; Figure 5E), entirely corroborating our cell-based experiments. Thus, the data strongly support a model whereby binding of clients to HSP70 triggers interaction with UBQLN2, which then bridges binding to the proteasome to mediate degradation. For disease-linked UBQLN2, mutations no longer support interaction with client-bound HSP70 and aggregate clearance is impaired.

HSP70 can be roughly divided into two distinct domains, the N-terminal ATPase domain and the C-terminal substrate-binding domain, where also regulatory proteins such as the ubiquitin ligase CHIP bind (Zhang et al., 2015). We found that the C terminus of HSP70 is sufficient to bind UBQLN2, but unlike for the full-length protein, the interaction was constitutive and not regulated by heat shock (Figure 5F). We also tested if the PXXP motif is required for interaction; however, deletion of the PXXP motif had no effect on HSP70 binding, demonstrating that this region is not the direct binding site (Figure S5G). Instead, it is likely that the PXXP mutations interfere indirectly with HSP70 binding.

### UBQLN2 Mutation Leads to Cognitive Impairment and Inclusion Body Pathology in Mice

After confirming decreased UBQLN2-HSP70 binding in knockin mouse brain (Figure 5G), we undertook a longitudinal behavioral study to determine the effect on mouse behavior. Using novel object recognition tests, where the time that a mouse spends exploring a novel versus familiar object is measured, we observed that mutant UBQLN2 (mP520T) animals were no longer able to distinguish between novel and familiar objects at 12 months of age (Figure 5H). Similarly, in novel place recognition tests (Figure 5I), mutant animals were incapable of distinguishing an object in a new location at both 9 and 12 months of age. Thus, UBQLN2 (mP520T) knockin mice develop cognitive deficits with age.

**Figure S7:**
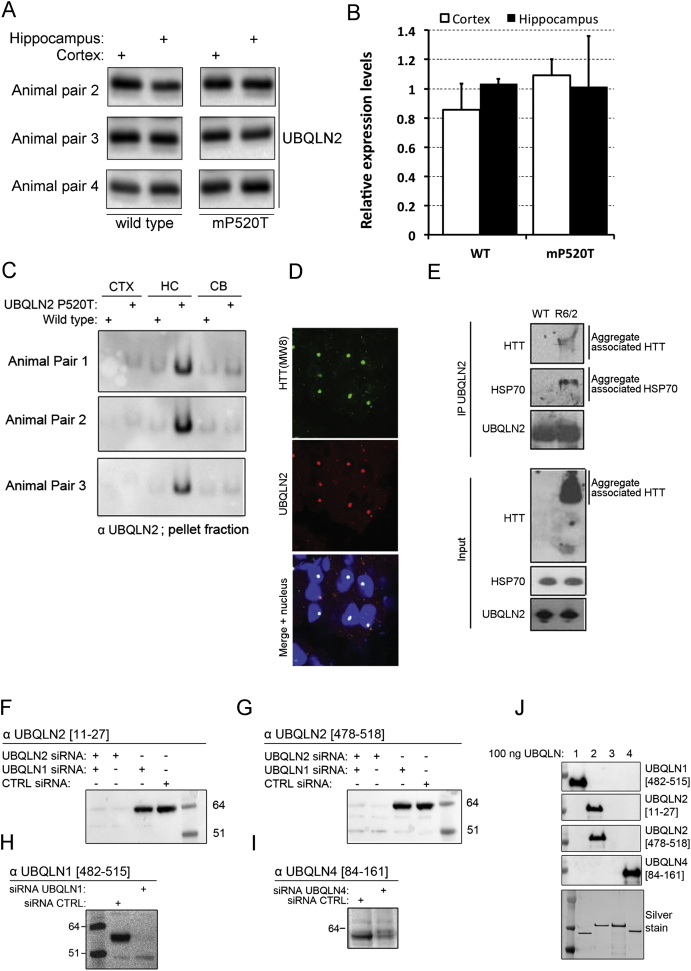
UBQLN2 Is Aggregated in Hippocampus and Associates to HSP70 and HTT Aggregates; Characterization of UBQLN Antibodies, Related to Figures 1, 2, 4, 5, and 6 (A and B) Hippocampal or cortical extracts were examined from WT or P520T knock-in mice. UBQLN2 expression levels were quantified and found to be indistinguishable between brain regions in either genotype. (C) UBQLN2 was found in the pellet fraction of the hippocampus in P520T knock-in, but not WT mice, in three additional independent pairs of animals. (D) UBQLN2 co-localizes with HTT inclusions in R6/2 brains. Sections of 14 week R6/2 brains were stained for HTT (MW8 antibody) and UBQLN2. (E) UBQLN2 was immunoprecipitated from 14-week-old R6/2 brains and blotted for the indicated proteins. HTT and HSP70 were detected in the stacking gel, indicated UBQLN2 interacts with SDS-insoluble HTT aggregates that are positive for HSP70. (F–I) Validation of specificity for UBQLN antibodies produced in-house. All antibodies were raised in sheep. UBQLN1, UBQLN2 or UBQLN4 were knocked down using siRNA and the indicated antibody used for detection. No cross-reactivity between ubiquilins was seen. (J) Purified untagged mouse UBQLN1, 2, 3 and 4 was further used to assess specificity of the raised antibodies, which confirm that there is no cross reactions.

As patients also have motor defects, we tested the UBQLN2 (mP520T) knockin animals using gait and rotarod analysis (Figures S6C–S6E) but observed no gross defects in either assay, although mutant mice presented with a slightly shorter stride length (Figure S6C). To assess if the cognitive deficits were accompanied by pathological changes, we performed immunohistochemical analyses on CNS tissues from 15- to 18-month-old mice. We observed regionalized UBQLN2 and p62 inclusion pathology in the hippocampus, cortex, and brainstem of mutant, but not WT, mice (Figure 5J). Interestingly, UBQLN2 is prominently present in the pellet fraction in hippocampal, but not cortical or cerebellar, tissue, despite similar expression levels (Figure 5K; Figures S7A and S7B). Importantly, our combined behavioral and histological findings demonstrate that UBQLN2 (mP520T) knockin mice recapitulate cognitive and pathological features of UBQLN2-associated neurodegeneration.

### UBQLN2 Mutation Impairs the Clearance of Protein Aggregates In Vivo

**Figure 6:**
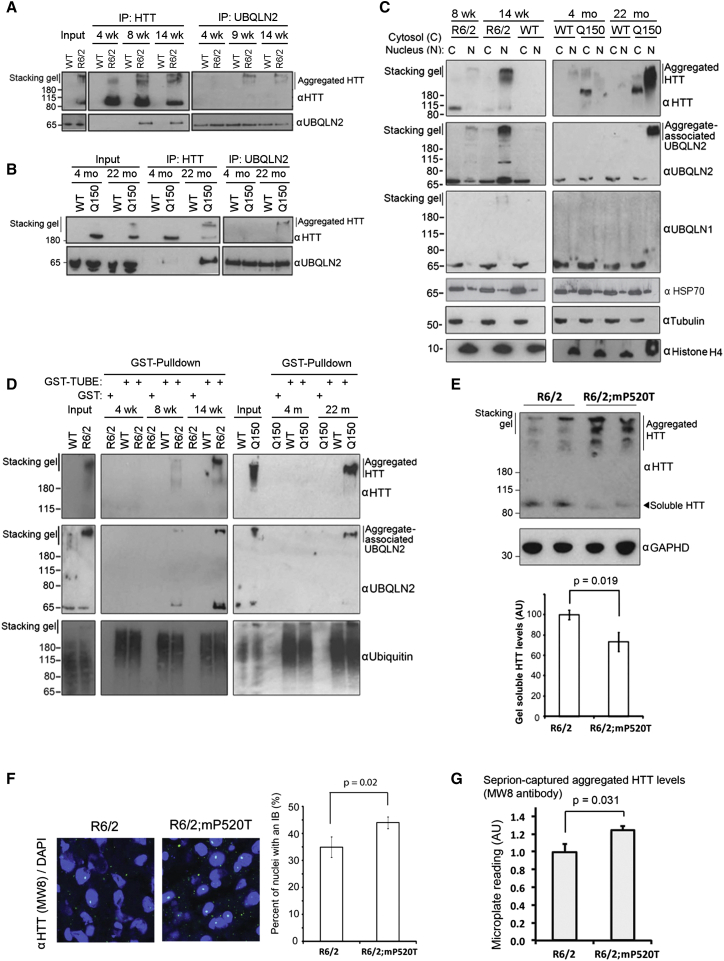
UBQLN2 Mutation Impairs Aggregate Clearance In Vivo (A and B) UBQLN2 interacts with aggregated, but not SDS-soluble, HTT in vivo, as judged by reciprocal IP of HTT and UBQLN2 from the R6/2 transgenic (A) and *Hdh*Q150 knockin (B) Huntington’s disease models. (C) UBQLN2, but not UBQLN1, translocates to the nucleus in the R6/2 and *Hdh*Q150 models. (D) UBQLN2 is present in ubiquitylated Huntingtin aggregates from brains of the R6/2 and *Hdh*Q150 mouse models. Aggregated HTT and UBQLN2 were captured with a ubiquitin binding resin (GST-TUBE). (E) The R6/2 and UBQLN2 mP520T mice were crossed to produce double-mutant animals, and 9-week-old male brains from these were assayed for aggregated HTT by western blot. Quantification of soluble HTT is shown (bottom) (n = 4 per genotype). (F) Immunofluorescence (IF) of nuclear HTT aggregates in R6/2 and R6/2;mP520T brains shows more inclusion bodies in the double mutant. Quantification is shown (right). Error bars represent SEM. Statistical test was a two-tailed t test. (G) The Seprion ligand assay independently confirms a significant increase in aggregated HTT in double mutants, compared to R6/2 littermates (n = 8 per genotype). Error bars represent SEM. See also Figure S7.

To examine the role of UBQLN2 in handling aggregating clients in vivo, we turned to mutant Huntingtin (HTT) as a representative model. Using the R6/2 transgenic mouse (Mangiarini et al., 1996) and the *Hdh*Q150 knockin mouse model (Lin et al., 2001), we found that immunoprecipitated UBQLN2 only associated with aggregated, but not SDS-soluble, HTT in vivo (Figures 6A and 6B). In both mouse models, binding of UBQLN2 to HTT was age- and disease-stage specific and only occurred once HTT had aggregated. HTT fragments passively diffuse into the nucleus in neurons, where they are retained upon aggregation (Cornett et al., 2005). Importantly, nuclear aggregation of HTT in both mouse models led to a translocation of UBQLN2, but not UBQLN1, to the nucleus (Figure 6C; Figure S7D). A proportion of HSP70 was present in nuclei at all ages (Figure 6C), and importantly, aggregate-associated HSP70 was trapped in the stacking gel in UBQLN2 immunoprecipitations from R6/2 brains (Figure S7E). Thus, mouse UBQLN2 behaves identically in HD mouse brains to UBQLN2 in cultured cells after heat shock.

Moreover, UBQLN2 and HTT were co-captured by a ubiquitin binding resin (Hjerpe et al., 2009) (TUBE; Figure 6D), demonstrating that HTT-UBQLN2 complexes contain ubiquitin, suggesting they may be cleared by the proteasome.

To directly test if UBQLN2 regulates HTT aggregation in vivo, we crossed R6/2 mice with UBQLN2 mP520T mutant knockin mice and observed a pronounced and significant increase of aggregated HTT, and a concomitant decrease of soluble HTT (Figure 6E). UBQLN2 co-localized with HTT inclusions (Figure S7D), and the number of nuclear HTT aggregates was significantly higher in the cortex of R6/2; mP520T double mutant animals compared to the R6/2 animals (Figure 6F). Moreover, a Seprion ligand assay shows significantly higher aggregate load in double-mutant brains, independently confirming our western blot and immunofluorescence analysis (Figure 6G). Thus, UBQLN2 mediates the clearance of protein aggregates in vivo, and the disease-linked forms of UBQLN2 are loss-of-function mutations, resulting in a failure to clear aggregating proteins.

## Discussion

### Proteasome Shuttle Factors as a Route for Protein Degradation

Degradation through the UPS is the major cellular mechanism of selective protein turnover. We have shown that the shuttle factor UBQLN2 works with the HSP70 system for proteasomal degradation of insoluble ubiquitylated protein aggregates. UBQLN2 does this by coupling recognition of HSP70-bound clients with its proteasome shuttle properties. UBQLN2 binding to ubiquitylated proteins and the proteasome is negligible under resting conditions, suggesting it is constitutively held in an inactive state. Accumulation of clients results in an activation of UBQLN2, mediated by recognizing client-bound HSP70, where binding to ubiquitylated substrates is induced and degradation facilitated.

### UBQLN2 Integrates the Chaperone Network with the UPS to Clear Protein Aggregates

UBQLN2 is needed both for aggregate clearance and survival after proteotoxic stress, suggesting that it is an integral component of the proteostasis network similar to HSP70 (Labbadia and Morimoto, 2015). Our finding that efficient binding of UBQLN2 to HSP70 requires the presence of HSP70 clients integrates the chaperone network with the UPS.

**Figure 7:**
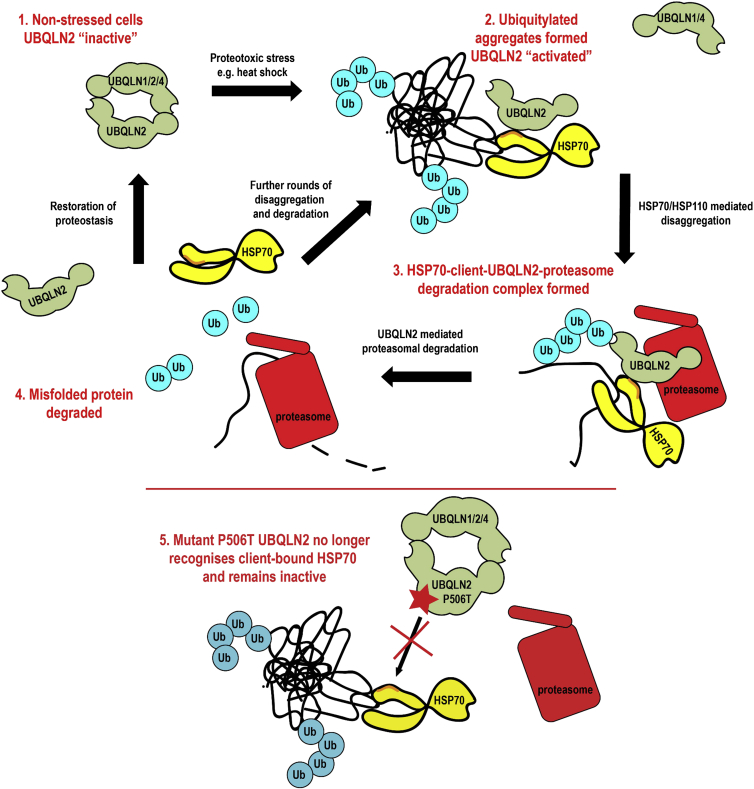
Model of How UBQLN2 Manages Proteotoxic Stress Under non-stressed conditions, UBQLN2 is held inactive in homo- or hetero-dimers (1). In the presence of HSP70 clients, UBQLN2 binds to HSP70 and associated misfolded/aggregated proteins, which are ubiquitylated (2). HSP70/HSP110-dependent disaggregase activity pulls aggregated proteins apart, allowing for UBQLN2 to act as a proteasome shuttle connecting ubiquitylated misfolded proteins to the proteasome, after forming a HSP70-client-UBQLN2-proteasome degradation complex (3) ending in client proteolysis (4). Disease mutant UBQLN2 (star) is defective in its association to HSP70 and no longer effectively forms a degradation complex, leading to accumulation of misfolded/aggregated proteins (5).

Our conclusions are summarized in Figure 7. Briefly, under resting conditions, UBQLN2 is inactive and bound to other UBQLNs and itself. Activation of UBQLN2 occurs when HSP70 binds to client proteins, triggering exposure of a UBQLN2 binding site. A structural change in HSP70 mediated by client binding would provide efficient and fast means of activating degradation, while ensuring that complexes are only formed in the presence of unfolded client. Activation of UBQLN2 also allows binding of 26S proteasome to form a degradation-competent complex. Interestingly, initial complex formation among client-bound HSP70, UBQLN2, and proteasome does not require polyubiquitylation of the client. However, ubiquitin is an integral part of proteasomal degradation, and heat-shock-induced aggregated proteins are ubiquitylated. Ubiquitylation of an HSP70 client could thus take place with UBQLN2 already present in the complex and may enhance UBQLN2 affinity, committing the client to proteasomal degradation. This model explains why we observe the inducible binding of UBQLN2 to ubiquitylated proteins after heat shock. Moreover, it is very likely that translocation into the proteolytic chamber and degradation of the substrate by the proteasome requires polyubiquitylation of the client, even though initial complex formation does not. Whether a client is refolded by HSP70 or degraded by UBQLN2/UPS may ultimately be a question of its residence time on HSP70.

### The HSP70-UBQLN2-Proteasome Pathway Provides an Autophagy-Independent Means for Clearing Protein Aggregates

Since proteasomes can only accommodate single unfolded polypeptide chains and not large aggregates, it has been assumed that the proteasome cannot degrade these. We demonstrate that the proteasome can clear aggregates through a UBQLN2-HSP70 pathway but suggest aggregates are first solubilized by HSP70-HSP110 disaggregase activity. Lending support to this idea, we show that the HSP70 cofactor HSP110, which is part of the HSP70-mediated disaggregase (Nillegoda et al., 2015), is also required for the efficient clearance of heat shock aggregates. UBQLN2 likely binds to HSP70 associated to both insoluble and soluble misfolded proteins as part of an ongoing disaggregation and clearance activity, which explains our observation that UBQLN2 co-purifies with insoluble ubiquitylated aggregates. This model is consistent with previous reports that demonstrate that aggregates exist in equilibrium between soluble and insoluble states (Yamamoto et al., 2000), and we propose that the soluble fraction is degraded by the proteasome, while autophagy may manage larger insoluble structures. Critically, we show that UBQLN2 can clear aggregates in the nucleus, where autophagy is absent (Lu et al., 2014).

### UBQLN2 Loss-of-Function Mutations Lead to Disease Due to Loss of HSP70 Binding

It has been unclear whether UBQLN2 mutations cause disease through loss of function or toxic gain of function. We found that a disease-linked mutation led to a pronounced sensitivity to proteotoxic stress, effectively phenocopying the effect of UBQLN2 depletion, strongly suggesting a loss-of-function mutation. Our data demonstrate that this defect is due to impaired interaction with HSP70, ultimately leading to defective aggregate clearance (Figure 7). Interestingly, translocation of UBQLN2 into the nucleus was not affected by the disease mutation, suggesting that this aspect of the stress response is independent of HSP70 binding. This makes sense, as our model predicts that activation of UBQLN2 would rely upon association to client-bound HSP70, and it is unlikely that such a complex would be formed in the cytoplasm and then driven into the nucleus. However, the mechanism by which UBQLN2 is tranlsocated into the nucleus as inactive species is currently unclear.

We also found that the mutant form of UBQLN2 binds slightly more polyubiquitin than the WT under unstressed conditions. The reason is not apparent, but it may be due to UBQLN2 occasionally dissociating from its inactive state under resting conditions, leading to binding to polyubiquitylated proteins and a possible delay of mutant UBQLN2 in returning to its inhibited state. This difference is dramatically swamped under stress conditions, where ubiquitin binding by mutant UBQLN2 is significantly decreased versus the WT protein.

Together, our data provide a mechanistic understanding of UBQLN2, which in the future may allow for the design of small molecules to mediate the therapeutic activation of UBQLN2 in patients with diseases of protein aggregation.

## Experimental Procedures

### Animal Work

UBQLN2 P520T constitutive knock-in mice were created and supplied by Taconic/Artemis. R6/2 mice were maintained as previously described (Bett et al., 2006). Mice were bred at the University of Dundee and Kings College London in accordance with European Union and Home Office regulations. Work was approved by the Ethical Review Committee (ERC) from the University of Dundee and was performed with a UK Home Office project license. R6/2 males were bred with heterozygous UBQLN2 P520T females at Charles River Laboratories (UK).

### Cell Culture and Cell Lines

Cells stably expressing inducible FLAG-UBQLN2 WT, P506T, P497H, L619A, P506T/L619A, P497H/L619A, HTTQ25-GFP, and HTTQ103-GFP were created using T-Rex HEK293 (Life Technologies, R710-07). Stably expressing cells were maintained in DMEM (Life Technologies, 11995-065), 10% fetal bovine serum (FBS), 50 U/ml penicillin, 50 μg/ml streptomycin (Life Technologies, 15070-063), 2 mM L-glutamine, 100 μg/ml hygromycin (Invivogen, ant-hg-1bl), and 15 μg/ml blasticidin (Invivogen, ant-bl-1). Expression was induced with 2–5 ng/ml doxycycline. U2OS cells, HEK293 cells, and MEFs were maintained as above but without hygromycin and blasticidin.

### Solubility Experiments

Cells were heat shocked at the indicated temperature for 2 hr followed by recovery at 37°C. Soluble and pellet fractions were generated by lysing cells in stringent lysis buffer (20 mM Tris-HCL, 2 mM EDTA, 150 mM NaCl, 1.2% deoxycholate, 1.2% Triton-X, 200 mM iodoacetamide and cOmplete protease inhibitor cocktail [Roche]), sonicating (30% power 3 × 10 s pulses), and centrifugation at 17,000 × *g* for 15 min. The supernatant was collected and represented the soluble fraction. The remaining pellet (insoluble fraction) was washed five times in PBS and re-suspended in Laemmli’s sample buffer. To generate the cytosolic-soluble, nuclear-soluble, and total-insoluble fractions, cells were first lysed in low-stringency buffer (10 mM HEPES [pH 7.9], 1.5 mM MgCl_2_, 10 mM KCL, 0.08% NP-40, and cOmplete protease inhibitor cocktail [Roche]) followed by centrifugation at 17,000 × *g* for 15 min. The supernatant (soluble fraction) was collected. The remaining pellet was washed five times in PBS prior to re-suspending in stringent lysis buffer, and soluble and insoluble fractions were generated as above. In this case, the supernatant represented the nuclear-soluble fraction and the pellet represented the total-insoluble fraction.

### Cell Viability Assays

Cell viability assays were done by lysing cells in 50 mM Tris/phosphate (pH 7.8), 1.6 mM MgCl_2_, 2 mM DTT, 2% Triton X-100, 30% glycerol, 1% BSA, 0.250 mM D-luciferin, 8 μM sodium pyrophosphate, and 500 ng QuantiLum recombinant Luciferase (Promega). Viability was determined using Envision 2104 plate reader (Perkin Elmer). Cells were heat shocked for 2 hr followed by 24 hr recovery prior to viability assay being carried out.

### Antibodies

Sheep antibodies to UBQLN1, UBQLN2, and UBQLN4 were produced in house, raised against the following epitopes (residues numbered): mouse UBQLN1 482-515, mouse UBQLN2 11-27, human UBQLN2 478-518, mouse UBQLN4 84-161 (Figures S7F–S7J). Additional antibodies were FLAG-M2-peroxidase (Sigma-Aldrich, A8592), HSP70 (Abcam, ab181606), GAPDH (Cell Signaling Technology), Actin (Millipore, MAB1501R), anti-ubiquitin (Dako, Z 0458), GFP (Roche), Histone H4 (Abcam), histone H3B (Abcam), HTT (Bett et al., 2006), tubulin (Sigma), RPT6 (Enzo Life Sciences, BML PW9265), puromycin 12D10, (Millipore, MABE343). For immunofluorescence, anti-UBQLN2 from Novus Biologicals (NBP2-25164SS), anti-RPT3 (Bethyl Laboratories, A303-850A), and anti-GFP (Abcam, ab13970) were used. Secondary antibodies were from Bio-Rad (anti-mouse 170-5047; anti-rabbit 170-5046) and Abcam (anti-sheep ab97130). Protein-G horseradish peroxidase (HRP) was used for secondary detection in immunoprecipitations (Abcam, ab7460).

## Author Contributions

R.H. and J.S.B. performed all of the experiments described with the exception of the biophysical experiments (analytical ultra centrifugation [AUC], circular dichroism [CD], and SAXS), which were performed by A.S., and the Seprion Ligand assay, which was performed by G.O. and G.B. A.K. and C.J. set up conditions for purification of UBQLN2. R.H. purified UBQLN2 for AUC, CD, and SAXS experiments. M.J.K. performed immunofluorescence staining of endogenous UBQLN2 after heat shock and of HTTQ103 cells. T.G.M. performed cardiac perfusions and mouse brain sub-dissections. F.M. performed IHC on mouse brains and analyzed pathology. I.S. purified human proteasome and aided in the characterization of in vitro complexes between UBQLN2 and the 26S proteasome with advice from M.H.G. A.K. provided all other purified proteins. M.T. and J.V. performed the mass spectrometry analyses. N.W. and M.W. generated cDNA clones. R.H. initiated the work on the effect of UBQLN2 mutations in vitro and in vivo and established the UBQLN2 interactome and its role in newly synthesized protein stress. J.S.B. initiated the work on the concept of UBQLN2 as a stress-activated proteasome shuttle in the clearance of heat-induced protein aggregates and in the in vivo clearance of HTT. R.H., J.S.B., and T.K. designed, interpreted, and analyzed the experiments and wrote the paper with contributions from all the other authors. T.K. conceived the project and supervised the work.

## Supplemental Information

Document S1. Supplemental Experimental Procedures

Document S2. Article plus Supplemental Information
