# Supplementary material for: UBQLN2 Mediates Autophagy-Independent Protein Aggregate Clearance by the Proteasome
Source: Cell. 2016 Aug 11;166(4):935–49. doi: 10.1016/j.cell.2016.07.001 (PMC5003816; doi:10.1016/j.cell.2016.07.001)
Supplement: Document S1. Supplemental Experimental Procedures [file mmc1.pdf]

Cell, Volume *166*

Supplemental Information

UBQLN2 Mediates Autophagy-Independent Protein Aggregate Clearance by the Proteasome

Roland Hjerpe, John S. Bett, Matthew J. Keuss, Alexandra Solovyova, Thomas G. McWilliams, Clare Johnson, Indrajit Sahu, Joby Varghese, Nicola Wood, Melanie Wightman, Georgina Osborne, Gillian P. Bates, Michael H. Glickman, Matthias Trost, Axel Knebel, Francesco Marchesi, and Thimo Kurz

Cell, Volume *166*

Supplemental Information

UBQLN2 Mediates Autophagy-Independent

Protein Aggregate Clearance by the Proteasome

Roland Hjerpe, John S. Bett, Matthew J. Keuss, Alexandra Solovyova, Thomas G. McWilliams, Clare Johnson, Indrajit Sahu, Joby Varghese, Nicola Wood, Melanie Wightman, Georgina Osborne, Gillian P. Bates, Michael H. Glickman, Matthias Trost, Axel Knebel, Francesco Marchesi, and Thimo Kurz

Extended Experimental Procedures

Mouse brain tissue lysis

Mouse brains were dissected out and immediately frozen in liquid nitrogen. UBQLN2 brain lysates were prepared by homogenizing one brain in 3 ml CHAPS buffer (50 mM TRIS pH 7.5, 0.3 % w/v CHAPS (SIGMA ALDRICH C9426), 270 mM sucrose (VWR 27480.360), 10 mM chloroacetamide (SIGMA ALDRICH C0267), cOmplete protease inhibitor cocktail (Roche 04693159001) and phosSTOP phosphatase inhibitor cocktail (Roche 04906837001)). For HTT mouse brains, lysates were homogenized in NP-40 buffer (50 mM HEPES/KOH pH 7.2, 400 mM NaCl, 1% NP-40, 0.2 mM EDTA, 10% glycerol, 100 mM iodacetamide and cOmplete protease inhibitor cocktail (Roche 04693159001). Lysates were clarified by centrifugation at 10,000 g at 4°C for 30 min. To generate nuclear and cytoplasmic preparations from mouse brain, brain hemispheres were homogenized in 750 µl sucrose "buffer 1" (575 mM sucrose, 25 mM KCl, 50 mM Triethanolamine, 5 mM $MgCl_2$, 1 mM DTT and protease inhibitor cocktail) with a Dounce homogenizer and the sample centrifuged at 800 g for 15 minutes. The supernatant fraction (cytoplasmic) was removed and stored at -80°C. The pellet fraction was resuspended in 1.5 ml sucrose "buffer 1", followed by an addition of two volumes of sucrose "buffer 2"(2.3 M sucrose, 25 mM KCl, 50 mM Triethanolamine, 5 mM $MgCl_2$, 1 mM DTT and protease inhibitor cocktail) and mixed gently by inversion. The re-suspended pellet fraction was carefully layered onto a sucrose cushion (0.5 ml of "buffer 2") in Beckman SW41 rotor tubes and centrifuged at 124,000 g for 1 h in a Beckman Ultracentrifuge. The supernatant was discarded, and nuclei were re-suspended in 100 µl "buffer 1" These were washed three times and re-suspended in 100 µl "buffer 1" and mixed with sample buffer before loading for western analysis.

IP for mass spectrometry to isolate UBQLN2 binding partners

Brain lysates were pre-cleared by incubation for 20 minutes at 4°C with 100 µl agarose beads per 50 mg total protein, repeated three times. 500 µg antibody was captured to 100 µl protein-G agarose resin for 3.5 hours at 4°C. Captured antibodies were washed twice with 1 ml PBS. For crosslinking, antibodies were washed three times in 1 ml 0.1 M sodium borate pH 9.3. The resin was then incubated rotating in 20 mM Dimethyl pimelimidate, 0.1 M sodium borate pH 9.3 at room temperature for 20 minutes, repeated two times. The crosslinked antibodies were then washed four times 1 ml 50 mM glycine pH 2.5 and twice in 200 mM TRIS pH 8, and finally re-suspended in 200 mM TRIS pH 8 prior to use. For immunoprecipitation of UBQLN2, 500 µg of antibody raised to either amino acids 11-27 or 478-518 of UBQLN2 were used per 20 mg total brain lysate. Immunoprecipitations were done for 1.5 hours at 4°C. Beads were washed four times 1 ml with cold CHAPS buffer (see above), supplemented with 150 mM NaCl, followed by transfer in cold PBS to a new Eppendorf tube to limit detergent carryover, and washed three times 1 ml PBS. Proteins were eluted by incubation with 200 µl 1:1 acetonitrile:1% formic acid, repeated five times, filtered to remove trace amount of beads and pooled. The eluate was then dried down in a vacuum concentrator and kept at -20°C until ready for sample processing.

SILAC proteomics

T-Rex^{TM} HEK 293 cells stably expressing inducible FLAG-UBQLN2 wild type or P506T were labeled with either isotopically heavy or light amino acids. For heavy labeling, cells were grown for a minimum of seven doublings in SILAC DMEM (PIERCE 88420) supplemented with 10% dialyzed FBS (GIBCO 26400), 80.2 μg/ml $^{13}C_6$,$^{15}N_4$ L-arginine (SIGMA ALDRICH 608033), 140 μg/ml $^{13}C_6$,$^{15}N_2$ L-Lysine (SIGMA ALDRICH 608041), 181.8 μg/ml L-proline (SIGMA ALDRICH P0380), 50 U/ml penicillin, 50 μg/ml Streptomycin (Life Technologies 15070-063), 100 μg/ml hygromycin (Invivogen ant-hg-1bl) and 15 μg/ml blasticidin (Invivogen ant-bl-1). For light labeling, cells were grown in the same media as above but supplemented with 84 μg/ml L-arginine (SIGMA ALDRICH A5006) and 140 μg/ml L-lysine (SIGMA ALDRICH W384704), instead of their heavy counterparts. Label switching was performed in separate experiments. Expression of FLAG-UBQLN2 was induced with 2 ng/ml doxycycline (SIGMA ALDRICH D9891) for 24 hours, after which cells were harvested by scraping in ice cold PBS. Lysis was performed in CHAPS buffer (see above). Cell extracts were clarified by centrifugation at 10,000g for 10 minutes at 4°C and protein concentrations were measured by BCA assay (PIERCE 23225). Equal amounts of FLAG-UBQLN2 WT and P506T lysate was added separately to 30 μl anti-FLAG M2 magnetic beads (SIGMA ALDRICH M8823), and incubated rotating at 4°C for 1h in an Eppendorf tube. Supernatants were aspirated, and beads for FLAG-UBQLN2 WT and P506T were pooled using cold lysis buffer, followed by four washes in 1 ml cold lysis buffer. Beads were transferred to a fresh Eppendorf tube followed by four washes in 1 ml cold PBS. Bound proteins were eluted using 100 μl 1:1 acetonitrile:1% formic acid (SIGMA ALDRICH 271004 and F0507), repeated five times. Eluates were dried in a vacuum concentrator and kept at -20°C until sample processing.

Mass spectrometry

Mass Spectrometry data was acquired on an Orbitrap Velos Pro (Thermo) set up with an EasySpray source using a Thermo-Fisher PepMap reverse phase C_{18}, 3µm, 75µm x 50cm column coupled to a Thermo U3000RSL system running a gradient with a flow of 300 nl/min and eluted with a 150 min linear gradient of 97% solvent A (0.1% formic acid, 3% DMSO in H_2O) to 35% solvent B (80% acetonitrile, 0.08% formic acid, 3% DMSO in H_2O), followed by a rise to 99%B at 155 min. The data were acquired in the data-dependent mode, automatically switching between MS and MS-MS acquisition. Full scan spectra (m/z 400-1600) were acquired in the orbitrap with resolution R = 60,000 at m/z 400 (after accumulation to an FTMS Full AGC Target; 1,000,000; MSn AGC Target; 100,000). The 20 most intense ions, above a specified minimum signal threshold (2,000), based upon a low resolution (R = 15,000) preview of the survey scan, were fragmented by collision induced dissociation and recorded in the linear ion trap, (Full AGC Target; 30,000. MSn AGC Target; 5,000). Protein identification and quantification were performed using MaxQuant Version 1.4.0.12 (Cox and Mann, Nature Biotechnology, 2008) with the following parameters: stable modification carbamidomethyl (C); variable modifications oxidation (M), acetylation (protein N-terminus); quantitation labels with SILAC light or K8R10; maximum 5 modifications per peptide, trypsin as enzyme and 2 missed cleavages. Searches were conducted using a Uniprot-Trembl Mus musculus or Homo Sapiens database, plus common contaminants. Mass accuracy was set to 10 ppm for precursor ions and 0.5 Da for ion trap MS/MS data. Identifications were filtered at a 1% false-discovery rate (FDR) at the protein level, accepting a minimum peptide length of 7. Quantification

used only razor and unique peptides, and required a minimum ratio count of 2. “Re-quantify” and “match between runs” were enabled.

Purification of UBQLN2 from bacteria

E. coli BL21 were transformed with His_6-SUMO1-UBQLN2 WT or P497H, P506T, P509S, P525S and T487I cloned in pET15b. Colonies were picked, and 15 ml starter cultures inoculated over night at 37°C in LB medium supplemented with 100 μg/ml carbenicillin (Formedium CAR00025). Following this, one liter LB with 100 μg/ml carbenicillin was inoculated with 4 ml starter culture and incubated at 37°C until OD_{600} = 0.8, at which point the incubator temperature was dropped to 15°C and expression was induced with 100 μM IPTG (Formedium IPTG025). After 20 hours of induction, cells were harvested by centrifugation at 4000 rpm for 45 minutes at 4°C. Pelleted bacteria were re-suspended in 30 ml/liter culture of 50 mM TRIS pH 7.5, 250 mM NaCl, 0.5 mM EDTA, 0.5% Triton-X100, 1 mM DTT, 10 μg/ml Leupeptin and 1 mM Pefabloc and lysed by sonication. Lysates were clarified by centrifugation at 250,000g for 30 minutes at 4°C and incubated rotating with 1 ml Ni^{2+}NTA Sepharose™ (GE Healthcare Life Sciences 17-5268-01) for 2.5 hours at 4°C. Beads were washed five times 10 ml with 50 mM TRIS pH 7.5, 250 mM NaCl, 20 mM imidazole and 1 mM DTT and twice with 50 mM TRIS pH 7.5, 150 mM NaCl, 1 mM DTT. His_6-SUMO1-UBQLN2 was eluted with 2 ml 50 mM TRIS pH 7.5, 150 mM NaCl, 1 mM DTT and 400 mM Imidazole, in five fractions. Sample volume was reduced to 2 ml and protein concentration was measure using BCA assay (PIERCE 23225). His_6-SUMO1 was cleaved off using a 1:150 ratio of the SENP1 SUMO protease (produced in-house) overnight at 4°C. Cleaved UBQLN2 was further purified by gel filtration using a HiLoad 16/600 Superdex™ 200 pg (GE Healthcare

28-9893-35) in 50 mM TRIS pH 7.5 and 150 mM NaCl. For HALO and HALO-tagged UBQLN2, E. coli BL21 was transformed with GST-HALO-UBQLN2, and induced and lysed as described above. Clarified extract was incubated with 200 µl glutathione Sepharose (PIERCE 16100) per liter culture, rotating at 4°C for 2 hours. Beads were washed twice in 10 ml 50 mM TRIS pH 7.5, 250 mM NaCl and 1 mM DTT and twice in 50 mM TRIS pH 7.5, 150 mM NaCl and 1 mM DTT. Elution was performed by incubation with 250 µg PreScission protease over night at 4°C to cleave off GST.

Immunoprecipitations

For immunopreciptation of material from HTT mouse brain or heat shock cell experiments, magnetic protein-G beads were used (Dynabeads, Life technologies 10004D) to avoid the inappropriate isolation of aggregated proteins. For immunoprecipitation from HTT brains, 1 µg antibody was used to isolate protein complexes from 250 µg brain extract and incubated with 50 µl Dynabeads (50% slurry) overnight at 4°C. Beads were washed 5 times with lysis buffer and proteins eluted in Laemmli's sample buffer and analysed by western blotting. For immunoprecipitation of UBQLN2 for Western blot analysis of associated proteins, 6-10 µg UBQLN2 antibody was used with 50 µl magnetic protein-G Dynabeads (50% slurry), and immunoprecipitations were performed as above. MLN7243 was obtained from Active Biochem (catalogue number A-1384).

Seprion Ligand Assay

Aggregates were captured in Seprion ligand coated plates (Microsens) and detected using the MW8 mouse monoclonal antibody (1:4000) as described (Sathasivam et al., 2010).

Analytical ultracentrifugation

Sedimentation velocity (SV) experiments were carried out in a Beckman Coulter (Palo Alto, CA, USA) ProteomeLab XL-I analytical ultracentrifuge using interference optics. All AUC runs were carried out at the rotation speed of 20,000 rpm using AnTi50 rotor and an experimental temperature of 4°C. The sample volume was 400 µl and sample concentrations ranged from 0.3 and 4.6 mg/ml. The density and viscosity of the buffer (50 mM Tris, pH 7.5, 150 mM NaCl) at experimental temperature 4°C was calculated using the SEDNTERP program (http://sednterp.unh.edu/) (Laue, Shah, Ridgeway, & Pelletier, 1992). The partial specific volume of proteins was calculated as an additive sum of values of constituent amino acids using program SEDNTERP and converted to the experimental temperature (Durchschlag, 1986). Sedimentation velocity profiles were treated using size-distribution c(s) model implemented in the program SEDFIT (Schuck, 1998). In order to determine the mass of each species, the c(s) distribution was converted to c(M) distribution. Each peak on the distribution plot was integrated in order to obtain the weight-averaged values for sedimentation coefficient and molecular mass. Integrated values of sedimentation coefficient (s) obtained at experimental conditions were converted to the standard conditions (s20,w) (which is the value of sedimentation coefficient in water at 20°C).

Protein Disorder Prediction

Protein sequences were submitted to the PONDR web engine (www.pondr.com) using the neural network predictor VL-XT (Romero et al., 2001). Access to PONDR was provided by Molecular Kinetics (Indianapolis, IN).

Circular dichroism (CD) spectrometry

Circular dichroism spectra were measured using a Jasco J-810 spectropolarimeter. Bandwidth was set to 2 nm and the scan speed was 20 nm/min. A 0.02 cm pathlength cuvette was used for far-UV CD spectra measurements (from 250 to 185 nm). The measurements were carried out in 10 mM phosphate buffer (pH 7.5), 100 mM NaF. All spectra were obtained at 20°C. Ten consecutive scans were averaged to obtain the resulting spectra. Ellipticity signal was converted to a molecular CD units (Δε) (Kelly & Price, 2000). The spectra were fitted using the CDSSTR(Sreerama & Woody, 2004) program implemented in www.dichroweb.cryst.bbk.ac.uk/

Small-Angle X-ray scattering

SAXS data for UBQLN2 and its mutants (P497H and P506T) in 50 mM Tris, pH 7.5, 150 mM NaCl were collected on the beam line 2.1 at Diamond Light Source (Didcot UK). The scattering curves were recorded at a wavelength of 1 Å and sample-detector distance of 3.9 m covering the momentum transfer range of $0.013 < q < 0.4$ Å-1, where $q = (4\pi\sin\theta)/\lambda$ and 2θ is the scattering angle. Sample concentrations ranged between 5 mg/ml and 0.5 mg/ml (at least five sample concentrations were measured) and the experimental temperature was 4°C. Initial data processing and averaging was carried out according to the beam line protocol. Data were normalized to the buffer scattering and scaled for concentration using PRIMUS. The data were checked for radiation damage and concentration-induced aggregation. The estimation of SAXS

invariants (such as intensities at zero angles, radii of gyration, volume-of-correlation) and flexibility analysis for all samples were carried out using program ScÅtter (www.bioisis.net) (Rambo & Tainer, 2011), (Reyes, Schwartz, Tainer, & Rambo, 2014), (Durand et al., 2010).

Animal work

UBQLN2 P520T constitutive knock-in mice were created and supplied by Taconic/Artemis. R6/2 mice were maintained as previously described (Bett et al., 2006). Mice were bred at the University of Dundee and Kings College London in accordance with European Union and Home Office regulations. Work was approved by the Ethical Review Committee (ERC) from the University of Dundee and was performed with a UK Home Office project license. R6/2 males were bred with heterozygous UBQLN2 P520T females at Charles River Laboratories (UK).

Isolation of mouse embryonic fibroblasts (MEFs)

E12.5 embryos were decapitated, followed by removal of red organs. Heads were used for genotyping. Remaining tissue was minced using scalpels, followed by incubation in trypsin for 5 minutes. Trypsinised tissue was dissociated by repeated pipetting in DMEM (Life Technologies 11995-065) supplemented with 10% FBS, 50 U/ml penicillin, 50 µg/ml Streptomycin (Life Technologies 15070-063) and 2 mM L-glutamine. Cells were seeded in dishes and grown under standard conditions (37°C, 5% CO_2). Genotyping was performed using the primers below, which amplify a 345 bp fragment for the wild type and a 420 bp fragment for the constitutive knock-in. Fwd primer: 5'-TCA CAT CTA GAA GGG TTA GCT CC-3'. Rev. primer: 5'-AGT

GCT TCC TAA TAG CTG AGT CC-3'. MEFs were immortalized by continuous passage

Plasmid vectors, cDNAs and cloning

Human and mouse ubiquilin sequences were amplified from the following EST sources. Human UBQLN2 (Genbank NM_013444.3) was amplified as a BamH1-Not-1 flanked ORF from EST IMAGE clone 4543266. Mouse UBQLN1 (Genbank NM_026842.4) was amplified as a BglII-Not1 flanked ORF from EST IMAGE clone 4015641, mouse UBQLN2 (Genbank NM_018798.2) as a BamH1-Not1 ORF from EST IMAGE clone 5708544, mouse UBQLN3 (Genbank NM_198623.2) as a Sal1-Not1 ORF from RIKEN EST clone 4933400K24 and mouse UBQLN4 (Genbank NM_033526.2) as a BamH1-Not1 ORF from EST IMAGE clone 4236887. ORFs were then subcloned into a variety of bacterial and mammalian expression vectors. Bacterial expression was either from a modified version of pGEX6P-1 (GE Healthcare LifeSciences), expressing an N-terminal GST-C3-HALO-thrombin (created by sub-cloning a BglII-BamH1 flanked HALO-thrombin PCR product into the BamH1 site of pGEX6P-1), or from a His6-SUMO expression system, created by sub-cloning an Nco1-BamH1 flanked PCR product comprising His6-Human SUMO1 (Genbank BC053528.1; amplified from EST IMAGE clone 3452929) into Nco1-BamH1 sites of pET15b (Novagen). In this system the 5' cloning site is removed by deletion mutagenesis following sub-cloning to remove extraneous residues immediately downstream of SUMO. For mammalian expression, ORFs were sub-cloned into either modified versions of pCMV5 backbone or pcDNA5TM FRT/TO (Life Technologies) with N-terminal FLAG tags. All PCR reactions were carried out using KOD Hot Start DNA Polymerase (Merck Millipore, Darmstadt, Germany). All

full-length products or fragments were cloned into pSc-B (Agilent) and fully sequenced prior to further sub-cloning or manipulation. All mutations and deletions were made following the QuikChange method (Agilent), but using KOD Hot Start DNA Polymerase. DNA sequencing was performed by the Sequencing Service at the College of Life Sciences, University of Dundee (www.dnaseq.co.uk).

Filter trap assay

The filter trap assay was performed as previously described (Wanker et al., 1999).

TUBEs pulldowns

Mouse brain extracts were prepared in NP-40 buffer (50 mM HEPES/KOH pH 7.2, 400 mM NaCl, 1% NP-40, 0.2 mM EDTA, 10% glycerol, 100 mM iodacetamide and cOmplete protease inhibitor cocktail) and centrifuged at 13,000 x g for 30 min. Supernatant was isolated and 500 µg incubated with 10 µg GST or GST-TUBEs and 50 µl glutathione magnetic beads (50% slurry) overnight at 4°C. Beads were washed 5 times and proteins eluted in sample buffer, and analyzed by western blot analysis.

Immunofluorescence

Cells stably expressing inducible FLAG-UBQLN2 WT, P506T, P497H, L619A, P506T/L619A and P497H/L619A were induced for 24 hours using 5 ng/ml doxycycline. Cells were washed in PBS, and fixed for 10 minutes using 3.7% formaldehyde in PBS. Formaldehyde was quenched with 50 mM glycine in PBS. Cells were permeabilized with 0.25% Triton-X100 in PBS for 5 minutes, followed by 3 washes in PBS. Cells were blocked in 5% BSA in PBS for 20 minutes. Primary antibody was anti-FLAG (SIGMA ALDRICH F3165) used at 1/500 for 1h in 5%

BSA PBS. Secondary antibody was an Alexa Fluor 488 anti-mouse (Abcam ab150113) used at 1/1000 for 0.5 h in 5% BSA PBS. For immunofluorescence of endogenous UBQLN2, U2OS cells were fixed using cold (-20°C) methanol for 1 min, washed 3 times with PBS and stained with mouse monoclonal anti-UBQLN2 6H9 (Novus NBP2-25164), at 1:1000 in 2% BSA PBS for over night. Secondary antibody was goat Anti-Mouse DyLight 488 (Abcam ab96871). Images were captured with a Leica SP2 confocal microscope. For immunofluorescence of R6/2 HTT brains, frozen 15 μM coronal brain sections were taken on a cryrostat (Leica). Sections were fixed in 4% paraformaldehyde, blocked in 10% serum, and incubated with anti-HTT MW8 (1 : 500) or UBQLN2 (epitope 11-27, 1 : 1000) and alexa-conjugated secondary antibodies (Molecular Probes). Images were captured on a Zeiss Pascal Exciter confocal microscope. For co-localization of UBQLN2, HSP70 and the Proteasome, HEK-293 cells with inducible HTTQ103-GFP expression were plated on coverslips coated with Poly-D-lysine (50 μg/mL). Cells were induced for 72 hours with 2 μg/mL tetracycline, washed once with ice cold PBS and fixed with -20°C methanol for 1 min. After 3 washes with ice cold PBS, cells were blocked for 1 hour at RT with 3% BSA in PBS. Primary antibodies were chicken anti-GFP (Abcam ab13970, 1:5000), mouse anti-Ubqln2 (Novus Biologicals NBP2-25164SS, 1:1000), rabbit anti-Hsp70 (Abcam ab181606, 1:50) and rabbit anti-RPT3 (Bethyl Laboratories A303-850A, 1:1000), diluted in PBS with 3% BSA and incubated for 1 hour at RT with cells. Cells were washed 3 x 5 min with PBS and then incubated with secondary antibodies (anti-chicken Alexa Fluor 488, 1:2000, anti-mouse Alexa Fluor 647, 1:1000, and anti-rabbit Alexa Fluor 633, 1:500) for 1 hour at RT. Cells were washed 3 x 5 min with PBS and then stained with DAPI (0.2 μg/mL in PBS) for 5 min, washed once with PBS and then coverslips were mounted on slides with ProLong Gold Antifade (Thermo Fisher

Scientific). After drying, coverslips were visualized on a Zeiss LSM 710 confocal microscope.

Analytical gelfiltration

Purified UBQLN2 (see above) was subjected to chromatography over a Superdex 200 HiLoad 16/60 (GE-Healthcare Life Sciences), column to analyze oligomerization and aggregation.

siRNA experiments

Oligonucleotides were transfected using Lipofectamine RNAiMAX (Life Technologies 13778075) according to manufacturers instructions. Oligonucleotides for human UBQLN1 and UBQLN2 were silencer select from Ambion with siRNA ID s26831 (UBQLN1) and s26829 and s26828 (UBQLN2). HSPA1A (HSP70) siRNA was from Dharmacon (On-target plus). For mouse, s204092 (UBQLN1), s79752 and s79750 (UBQLN2) and s97078 (UBQLN4) were used. Oligonucleotides were used at a final concentration of 10 – 20 nM.

Proteasome activity probe experiments

Proteasome activity probe (Boston Biochem I-190) was used to determine activity of proteasome after heat shock. Cells heat shocked or kept at 37°C, followed by harvest and lysis in 50 mM TRIS pH 7.5, 5 mM $MgCl_2$, 250 mM sucrose, 1 mM DTT and 2 mM ATP. Cells were lysed by repeated aspiration through a 25 G needle using a syringe. Lysates were clarified by centrifugation at 4°C, and incubated with either DMSO (vehicle) or 50 μM MG132 for 30 minutes at 37°C. Proteasome activity probe was added to a final concentration of 1 μM, followed by incubation at 37°C for 1h.

Lysates were mixed with SDS sample buffer and resolved using standard SDS PAGE. Fluorescence was detected using a BioRad chemidoc XRS system. After fluorescent detection, gels were stained with Coomassie to control for equal loading.

Proteasome purification

Human 26S proteasomes were purified following a protocol adapted from (Glickman, Rubin, Fried, & Finley, 1998), with the modification that the source was human erythrocytes and the first separation step was on a DEAE column. LC-MS/MS assessment of final preparation confirmed presence of the canonical subunits viz., PSMA1-7, PSMB1-7, PSMC1-6 and PSMD1-14. No traces of HSP70 and UBQLN2 were found.

GST-pulldowns

GST-pulldown experiments were performed using 5 μM recombinant GST-HSP70, 5 μM recombinant UBQLN2 (WT or P520T) and 5 μM purified proteasomes where indicated. Pulldown reactions were incubated with either (1) 5 μM folded luciferase or luciferase that had been denatured at 42°C for 30 minutes or at 95°C for 5 minutes or (2) with 200 μg brain extracts from 14 week old WT or R6/2 mice. Pulldowns were performed using magnetic glutathione beads (Pierce) and washed five times stringently in buffer containing 50 mM HEPES pH 7.2, 400 mM NaCL, 1% NP-40, 0.2 mM EDTA and 10% glycerol before elution in sample buffer.

Behavioral phenotyping

Accelerating rotarod test was performed using a 5 lane Rota-Rod (Ugo Basilie). Testing was performed for 2 consecutive days, with 4 trials per day. The maximum trial time was set to 5 minutes, with an inter trial interval (ITI) of 30 minutes. The acceleration protocol was set from 5 to 45 rotations per minute, and latency to descent was recorded.

Fixed speed rotarod tests were performed using the same equipment as above, with one trial at each speed (10, 20, 30 and 40 rotations per minute), starting at the lowest speed. The maximum trial time was set to 5 minutes with an ITI of 1h. Latency to descent was recorded.

For gait analysis, mice were habituated to the walkway and to the scruffing procedure (2 days). For testing, front paws were painted red and hind paws were painted blue. Distance between paws and stride length was recorded.

Novel object recognition was performed in a white square arena 30 cm x 30 cm x 30 cm). Mice were habituated for two days in the empty arena (15 minutes per day) prior to testing. On the test day, mice were exposed to 2 identical copies of the same object (A^1 and A^2) in the sample phase, for a 10 minute trial time. This was followed by a 5 minute ITI, and the test phase, where the mice were exposed to 1 copy of a familiar object (A^3) and a novel object (B), with a trial time of 10 minutes. Activity was recorded by an overhead camera and behaviour tracked by AnyMaze software (Ugo Basilie). Time exploring the 2 objects and total time with objects were recorded.

Novel place recognition was performed in a white square arena (30 cm x 30 cm x 30 cm) with spatial cues on the walls. In the sample phase, mice were exposed to 2 identical copies of the same object (C^1 and C^2) with a trial time of 10 minutes. This was followed by an ITI of 5 minutes and the test phase, in which mice were exposed to 2 identical copies of the same object, with one of the objects in moved to a new

location within the arena. The trial time was set to 10 minutes and activity was recorded by an overhead camera and behaviour tracked by AnyMaze software (Ugo Basilie). Time exploring the two objects and total time with the objects was measured.

Tissue processing and immunohistochemistry

Aged mice were administered Euthetal via intraperitoneal injection and monitored throughout surgical anaesthesia. Upon a complete loss of responsiveness, animals were trans-cardially perfused at a constant rate with temperature-equilibrated phosphate buffered saline (pH 7.4). Subsequently, tissues (brain, thoracic spinal cord) were carefully excised and sub-dissected for downstream analyses. For histopathology studies, brains were hemisected along the superior sagittal sinus, and each left hemisphere was used for subsequent immunohistochemistry. Following 24 hours fixation the brain hemispheres were processed to paraffin block and microtome sectioning. Four μm sections were stained with Haematoxylin and Eosin (HE) for histological assessment. Additional serial sections were used for immunohistochemical analysis. Automated immunohistochemistry was performed with a Universal Staining System autostainer (DAKO). For p62/SQSTM1, heat induced epitope retrieval with 0.1M citrate buffer pH 6.0 in pressure cooker for 10 minutes was used. No epitope retrieval was applied to sections stained with anti-UBQLN2 antibody. The following primary antibodies were used: polyclonal rabbit anti-p62/SQSTM1 (MBL PM045) at 1:8000, polyclonal sheep anti-UBQLN2 (antibody raised in-house to amino acids 11-27 of mouse UBQLN2; the antibody was purified over the peptide antigen) at 1:400. The anti-rabbit ENVISION™ (DAKO) was used as visualisation system for p62/SQSTM1 antibody. For the UBQLN2 antibody, a secondary rabbit-anti sheep IgG followed by anti-rabbit ENVISION™

(DAKO) was used. Following reaction with DAB chromogen, slides were briefly rinsed in distilled water, followed by counterstain with Mayer's Haematoxylin for 30 seconds, rinsing in tap water, dehydration through ascending alcohol and xylene and coverslip mounting. All slides were assessed microscopically by a veterinary pathologist blinded to the group assignment and genotype of the animals. The presence and neuroanatomical location of inclusions/deposits staining with p62 and UBQLN2 was annotated. For biochemical studies, right hemispheres were acutely microdissected to isolate intact neural sub-regions of interest (hippocampus, cerebral cortex, cerebellum). Tissues were immediately snap-frozen in liquid nitrogen and stored at -80°C before processing.

Creation of the UBQLN2 P520T constitutive knock-in mouse model

The P520T, x-linked, constitutive knock-in mouse model was acquired from Taconic. The targeting strategy was based on NCBI transcript NM_018798.2, where exon 1 contains the complete open reading frame. The positive selection marker (Puromycin resistance - PuroR) has been flanked by FRT sites and inserted downstream of the Ubqln2 3' untranslated region (UTR). The targeting vector was generated using BAC clones from the C57BL/6J RPCIB-731 BAC library, and was transfected into the TaconicArtemis C57BL/6N Tac ES cell line. Homologous recombinant clones were isolated using positive (PuroR) and negative (Thymidine kinase - Tk) selection. The constitutive KI-PM allele was obtained after *in vitro* Flp-mediated removal of the selection marker. The remaining recombination site is located in a non-conserved region of the genome. Diploid injection: after administration of hormones, superovulated BALB/c females were mated with BALB/c males. Blastocysts were isolated from the uterus at dpc 3.5. For microinjection, blastocysts were placed in a drop of DMEM with 15% FCS under mineral oil. A flat tip, piezo actuated

microinjection-pipette with an internal diameter of 12 - 15 micrometer was used to inject 10-15 targeted C57BL/6NTac ES cells into each blastocyst. After recovery, 8 injected blastocysts were transferred to each uterine horn of 2.5 days post coitum, pseudopregnant NMRI females. Chimerism was measured in chimeras (G0) by coat color contribution of ES cells to the BALB/c host (black/white). Highly chimeric mice were bred to strain C57BL/6 females. Germline transmission was identified by the presence of black, strain C57BL/6, offspring (G1). Genotyping analysis: Genomic DNA was extracted from tail biopsies and analyzed by PCR. Genotyping PCR performed detects the constitutive Knock-In allele, after flpe-mediated deletion (IVD) of the puromycin resistance cassette, as well as the wildtype allele, using primers: Fwd: 5'-TCACATCTAGAAGGGTTAGCTCC-3', Rev: 5'-AGTGCTTCCTAATAGCTGAGTCC-3', with the expected fragment sizes (bp) 345(wt) and 420(const.ki). For detection of the introduced point mutation, the following primers were used: Fwd: 5'-CCATGTCAAACCCGAGAGC-3', Rev: 5'-TGAACTGCTGGTTGGGTCC-3'. Presence of the point mutations was confirmed in all mice by sequencing of the PCR product.

Supplementary references

Bett, J. S., Goellner, G. M., Woodman, B., Pratt, G., Rechsteiner, M., & Bates, G. P. (2006). Proteasome impairment does not contribute to pathogenesis in R6/2 Huntington's disease mice: exclusion of proteasome activator REGgamma as a therapeutic target. *Human Molecular Genetics*, *15*(1), 33–44. http://doi.org/10.1093/hmg/ddi423

Durand, D., Vivès, C., Cannella, D., Pérez, J., Pebay-Peyroula, E., Vachette, P., & Fieschi, F. (2010). NADPH oxidase activator p67(phox) behaves in solution as a multidomain protein with semi-flexible linkers. *Journal of Structural Biology*, *169*(1), 45–53. http://doi.org/10.1016/j.jsb.2009.08.009

Durchschlag, H. (1986). Specific Volumes of Biological Macromolecules and Some Other Molecules of Biological Interest. In *Thermodynamic data for biochemistry and ...* (pp. 45–128). Berlin, Heidelberg: Springer Berlin Heidelberg. http://doi.org/10.1007/978-3-642-71114-5_3

Glickman, M. H., Rubin, D. M., Fried, V. A., & Finley, D. (1998). The regulatory particle of the Saccharomyces cerevisiae proteasome. *Mol Cell Biol*, *18*(6), 3149–

3162.
Kelly, S. M., & Price, N. C. (2000). The use of circular dichroism in the investigation of protein structure and function. *Current Protein & Peptide Science*, *1*(4), 349–384.
Laue, T. M., Shah, B D, Ridgeway, T. M., & Pelletier, S. M. (1992). Analytical Ultracentrifugation in Biochemistry and Polymer Science. (A. Rowe & S. Harding, Eds.) (pp. 90–125). Redwood Press Ltd, Melksham, UK.
Rambo, R. P., & Tainer, J. A. (2011). Characterizing flexible and intrinsically unstructured biological macromolecules by SAS using the Porod-Debye law. *Biopolymers*, *95*(8), 559–571. http://doi.org/10.1002/bip.21638
Reyes, F. E., Schwartz, C. R., Tainer, J. A., & Rambo, R. P. (2014). Methods for using new conceptual tools and parameters to assess RNA structure by small-angle X-ray scattering. *Methods in Enzymology*, *549*, 235–263. http://doi.org/10.1016/B978-0-12-801122-5.00011-8
Romero, P., Obradovic, Z., Li, X., Garner, E. C., Brown, C. J., & Dunker, A. K. (2001). Sequence complexity of disordered protein. *Proteins*, *42*(1), 38–48. http://doi.org/10.1002/1097-0134(20010101)42:1<38::AID-PROT50>3.0.CO;2-3
Sathasivam, K., Lane, A., Legleiter, J., Warley, A., Woodman, B., Finkbeiner, S., et al. (2010). Identical oligomeric and fibrillar structures captured from the brains of R6/2 and knock-in mouse models of Huntington's disease. *Human Molecular Genetics*, *19*(1), 65–78. http://doi.org/10.1093/hmg/ddp467
Schuck, P. (1998). Sedimentation analysis of noninteracting and self-associating solutes using numerical solutions to the Lamm equation. *Biophysical Journal*, *75*(3), 1503–1512. http://doi.org/10.1016/S0006-3495(98)74069-X
Sreerama, N., & Woody, R. W. (2004). Computation and analysis of protein circular dichroism spectra. *Methods Enzymol*, *383*, 318–351. http://doi.org/10.1016/S0076-6879(04)83013-1
Wanker, E. E., Scherzinger, E., Heiser, V., Sittler, A., Eickhoff, H., & Lehrach, H. (1999). Membrane filter assay for detection of amyloid-like polyglutamine-containing protein aggregates. *Methods Enzymol*, *309*, 375–386.
